# Targeted Bmal1 restoration in muscle prolongs lifespan with systemic health effects in aging model

**DOI:** 10.1172/jci.insight.174007

**Published:** 2024-10-01

**Authors:** Miguel A. Gutierrez-Monreal, Christopher A. Wolff, Eduardo E. Rijos, Mark R. Viggars, Collin M. Douglas, Vishwajeeth Pagala, Junmin Peng, Liam C. Hunt, Haocheng Ding, Zhiguang Huo, Fabio Demontis, Karyn A. Esser

**Affiliations:** 1 Department of Physiology and Aging,; 2 Myology Institute, and; 3 Department of Molecular Genetics and Microbiology, University of Florida, Gainesville, Florida, USA.; 4 Department of Structural Biology, Center for Proteomics and Metabolomics, and; 5 Department of Developmental Neurobiology, St. Jude Children’s Research Hospital, Memphis, Tennessee, USA.; 6 Department of Biology, Rhodes College, Memphis, Tennessee, USA.; 7 Department of Biostatistics, University of Florida, Gainesville, Florida, USA.

**Keywords:** Muscle biology, Skeletal muscle

## Abstract

Disruption of the circadian clock in skeletal muscle worsens local and systemic health, leading to decreased muscle strength, metabolic dysfunction, and aging-like phenotypes. Whole-body knockout mice that lack *Bmal1*, a key component of the molecular clock, display premature aging. Here, by using adeno-associated viruses, we rescued *Bmal1* expression specifically in the skeletal muscle fibers of *Bmal1*-KO mice and found that this engaged the circadian clock and clock output gene expression, contributing to extended lifespan. Time course phenotypic analyses found that muscle strength, mobility, and glucose tolerance were improved with no effects on muscle mass or fiber size or type. A multiomics approach at 2 ages further determined that restored muscle *Bmal1* improved glucose handling pathways while concomitantly reducing lipid and protein metabolic pathways. The improved glucose tolerance and metabolic flexibility resulted in the systemic reduction of inflammatory signatures across peripheral tissues, including liver, lung, and white adipose fat. Together, these findings highlight the critical role of muscle *Bmal1* and downstream target genes for skeletal muscle homeostasis with considerable implications for systemic health.

## Abstract 2

Our findings underscore the critical role of the circadian clock in skeletal muscle function and metabolism and highlight the key role of skeletal muscle in systemic health and aging

## Graphical abstract

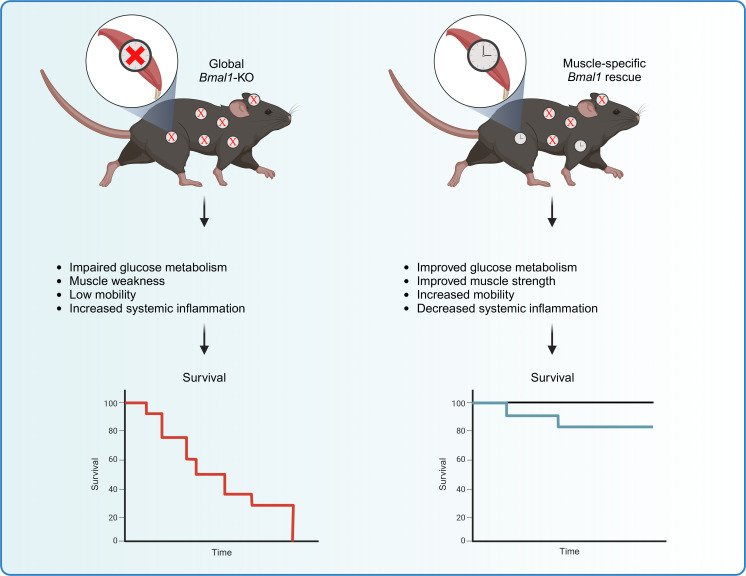

## Introduction

The circadian clock is a molecular timer found in virtually all cells of the body and contributes to the regulation of a daily program of gene expression influencing cell physiology (1). The positive arm of the clock is composed by the PAS domain helix-loop-helix transcription factors BMAL1 and CLOCK, which heterodimerize to activate the expression of the negative limb clock genes, *Period* (*Per1-3*) and *Cryptochrome* (*Cry1-2*). PER and CRY proteins heterodimerize, translocate to the nucleus, and inhibit the activity of BMAL1-CLOCK heterodimer, creating a cycle that takes about 24 hours to be completed (2, 3). The nuclear receptors ROR and NR1D1-2 comprise a secondary feedback loop that activates or represses, respectively, the expression of *Bmal1* gene by acting on ROR-binding elements within its promoter (4). In addition to timing, BMAL1-CLOCK contributes to cell and tissue homeostasis through the regulation of a daily transcriptional program, called clock output, that is important for cell-specific physiology and metabolism. While the core clock factors are common across cells, the clock output is tissue specific, and in skeletal muscle, clock output has been shown to be important for glucose metabolism as well as muscle structure and function (5–8).

Mice that lack expression of the *Bmal1* gene globally (*Bmal1*^–/–^ mice, herein referred to as *Bmal1*-KO mice) have been used as a model of premature aging because of their shortened median lifespan of approximately 37 weeks and various age-related health problems (9–11). An early study of *Bmal1* rescue in the *Bmal1*-KO mouse was focused on defining the tissue responsible for restoring rhythmic behavior (12). This study found that restoring *Bmal1* in the brain, but not skeletal muscle, was required for circadian behavior. While the authors did not test lifespan, they found that restoring *Bmal1* to skeletal muscle resulted in increased voluntary wheel activity, and they noted that those mice were not dying as early as the *Bmal1*-KO mice (12). Koronowski et al. in 2019 used a conditional gene trap approach to generate *Bmal1*-stop-FL mice (*Bmal1*-KO mice) that exhibited similar shortened lifespan (13). Using this *Bmal1*-stop-FL KO mouse, they rescued endogenous *Bmal1* expression in several tissues, including liver, brain, and epidermis, with no detectable effects on the survival of the *Bmal1*-stop-FL KO mouse (13–15). Most recently, Smith et al. in 2023 successfully restored the expression of *Bmal1* in the skeletal muscle of the *Bmal1*-stop-FL KO mouse and found an improvement in glucose metabolism. However, the impact on lifespan was not tested (16).

In this study we found that targeting the reexpression of *Bmal1* to skeletal muscle using an adeno-associated virus (AAV) delivery system in the global *Bmal1*-KO mouse was sufficient to markedly improve the lifespan in male mice. Using deep systemic and muscle phenotyping with multiomics analyses, we determined that expression of *Bmal1* from the AAV vector was sufficient to engage the core clock and key clock output genes in muscle, and this led to restoring muscle strength without affecting fiber size or type. Additional improvements include increased cage activity and improved glucose tolerance as well as metabolic flexibility accompanied by a decline in markers of systemic inflammation. The outcomes of this study highlight the important contributions of *Bmal1*, and likely key clock output genes, to healthy skeletal muscle. In addition, our findings point to the importance of healthy skeletal muscle for systemic health and lifespan.

## Results

### AAV9-mediated Bmal1 gene delivery into the skeletal muscle engages the muscle clock in global Bmal1-KO mice.

**Figure 1:**
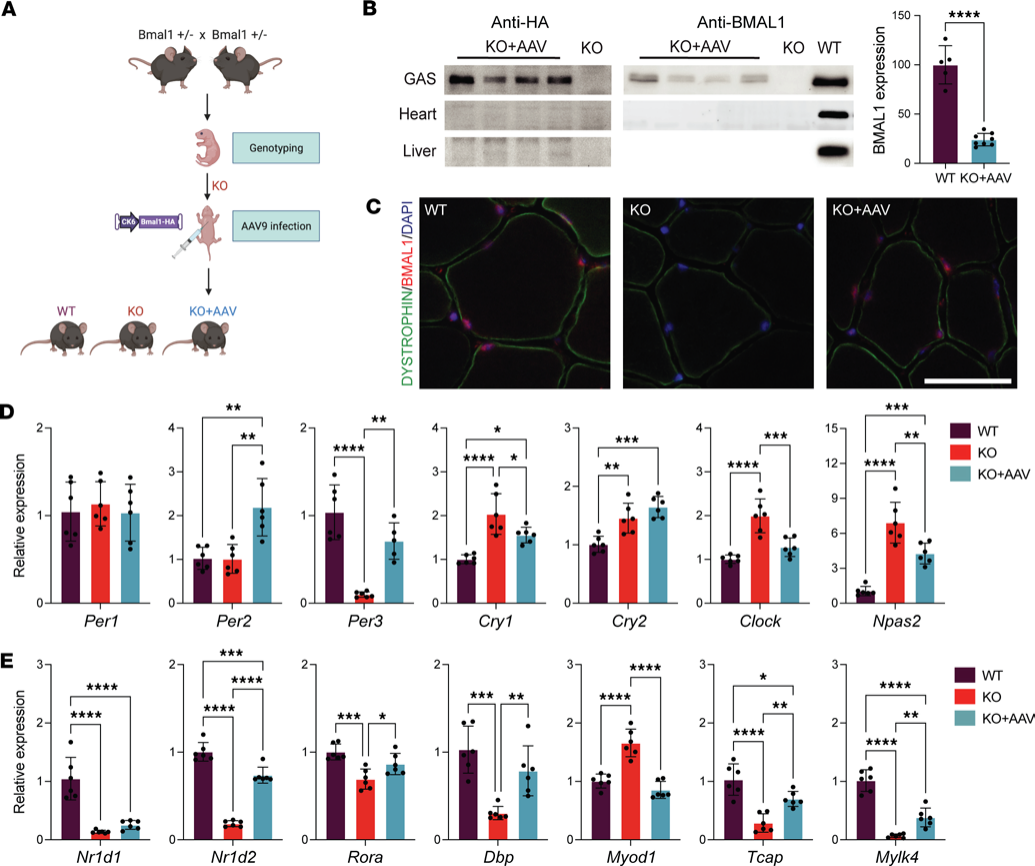
AAV-mediated *Bmal1* expression engages the muscle clock. (**A**) Schematic of the mouse generation workflow. *Bmal1*-KO^+/–^ mice were used as breeders. *Bmal1*-KO mice were genotyped at postnatal day 2–3 (P2–P3) and injected with AAV9 at P5. (**B**) Western blot for anti-HA and anti-BMAL1 detection in gastrocnemius (GAS), heart, and liver tissues from *Bmal1*-KO+AAV mice, demonstrating the muscle-specific rescue model at 10 weeks of age. (**C**) Representative images of BMAL1 immunostaining of tibialis anterior (TA) sections costained with dystrophin and DAPI from WT, *Bmal1*-KO, and *Bmal1*-KO+AAV mice. Scale bar = 50 μm. (**D**) Expression of core clock genes and (**E**) secondary clock and muscle output genes in gastrocnemius muscle (*n* = 5–6 mice/group). Data are shown as mean ± SD. One-way ANOVA with *P < 0.05, **P < 0.01, ***P < 0.001, ****P < 0.0001.

We systemically infected *Bmal1*-KO pups at postnatal day 5 with an AAV9-*Bmal1* vector that expresses hemagglutinin-tagged (HA-tagged) *Bmal1* cDNA under the control of the constitutive muscle-specific promoter, CK6 (Figure 1A). We first measured the protein levels of BMAL1 in the skeletal muscle of 10-week-old *Bmal1*-KO mice using antibodies against HA tag and BMAL1 (Figure 1B). We detected BMAL1-HA expression in gastrocnemius, tibialis anterior (TA), extensor digitorum longus (EDL), and quadriceps muscles whereas HA expression in soleus or diaphragm was undetectable, consistent with previous reports using the CK6 promoter (17, 18) (Supplemental Figure 1A; supplemental material available online with this article; https://doi.org/10.1172/jci.insight.174007DS1). We did not detect expression in the heart or liver, consistent with previous reports (17, 19, 20) (Figure 1B). We also tested lung and stomach samples, 2 tissues rich in smooth muscle cells, and we did not detect expression of HA (Supplemental Figure 1A). Using the BMAL1 antibody, we compared the level of expression of the transgene in KO+AAV with WT control muscle at a peak time for *Bmal1* expression (Zeitgeber time 1, ZT1). We found that in our mice we obtained on average approximately 25% of the expression levels found in WT muscles (range 16% to 34%). The expression of BMAL1 protein was also evident by immunohistochemical analysis of TA cryosections (Figure 1C). As expected, the expression of *Bmal1* in the KO+AAV muscle sections was detected in the muscle fiber nuclei within dystrophin staining, similar to WT muscle. Last, we compared the viral genome copy numbers in different muscle samples at 10 weeks of age, approximately 9 weeks after systemic infection, by real-time quantitative PCR (qPCR) (Supplemental Figure 1B). We found vector copies in each muscle tested. The TA, gastrocnemius, and quadriceps muscles had approximately 20,000 viral copies/μg genomic DNA, and the viral levels in the soleus were about 20 times lower. Liver and heart tissue had more copies than skeletal muscles, 28- and 6-fold compared with TA, respectively, but no protein was detected because the CK6 promoter is not active in those tissues (Figure 1B). These results validate the use of systemic delivery of AAV-CK6-*Bmal1*-HA as a model for skeletal muscle–specific *Bmal1* expression in the *Bmal1*-KO mouse.

We next asked whether the constitutive expression of BMAL1 from the CK6 promoter after birth is sufficient to engage the expression of core circadian clock factors as well as known clock output genes in muscle. Gastrocnemius muscles from 10-week-old WT, *Bmal1*-KO, and *Bmal1*-KO+AAV mice were collected at ZT1. We analyzed a total of 14 core clock factors and well-defined muscle-specific clock output genes by qPCR (Figure 1, D and E). For the core clock genes, we found that *Bmal1* transduction after birth was sufficient to reengage expression of most, but not all, of the core clock genes in the direction of WT muscle. This included upregulation of *Per3*, *Per2*, *Rora*, *Nr1d1*, and *Nr1d2* with downregulation of *Cry1*, *Clock*, and *Npas2* when compared with the expression in *Bmal1*-KO. Analysis of well-defined clock output genes showed that the expression levels of *Dbp*, *Tcap*, and *Mylk4* was upregulated while *Myod1* was downregulated relative to *Bmal1*-KO muscle. Thus, except for negative limb components (i.e., *Per1* and *Cry2*), rescuing the expression of *Bmal1* in the skeletal muscle of *Bmal1*-KO mice was sufficient to engage the expression of core clock components and of well-defined clock output genes similar to what is seen in WT muscle.

To address if the expression of AAV-*Bmal1* in the muscle of *Bmal1*-KO muscle was sufficient to induce a synchronized pattern of circadian clock gene expression, we compared gene expression at 2 time points, ZT1 and ZT13. At these time points, if the clocks were synchronized across all the cells in the muscle tissue, we would see time of day differences in the expression of *Per1*, *Per2*, *Per3*, *Cry2*, *Dbp*, and *Nr1d2* mRNAs (Supplemental Figure 1C). However, we did not see any notable time of day difference in core clock and clock output gene expression in the muscles of the KO+AAV mice, as seen in the WT muscle (Supplemental Figure 1C).

We next performed targeted ChIP-qPCR to determine if BMAL1 binds to known clock gene promoters in the KO+AAV muscle. As shown in Supplemental Figure 1D, we detected considerable enrichment of BMAL1 binding to the E-box–containing regions within the promoter of the core clock gene *Per2*, the clock output gene *Dbp*, and 2 muscle-specific clock output genes, *Tcap* and *Mylk4*, that we have shown previously to be bound by BMAL1 (Supplemental Figure 1D) (21). Overall, these results demonstrate that the reexpression of *Bmal1* in the *Bmal1*-KO muscle results in BMAL1 binding to known promoter elements and in changes in clock and clock output gene expression that are similar to WT muscle. However, the patterns of gene expression are not synchronized across the muscle, indicating that generation of a synchronized clock and clock output gene expression requires external cues, including information from an intact central clock in the suprachiasmatic nucleus.

### Skeletal muscle–specific expression of Bmal1 increases the lifespan of the Bmal1-KO mouse and improves mobility and glucose handling.

**Figure 2:**
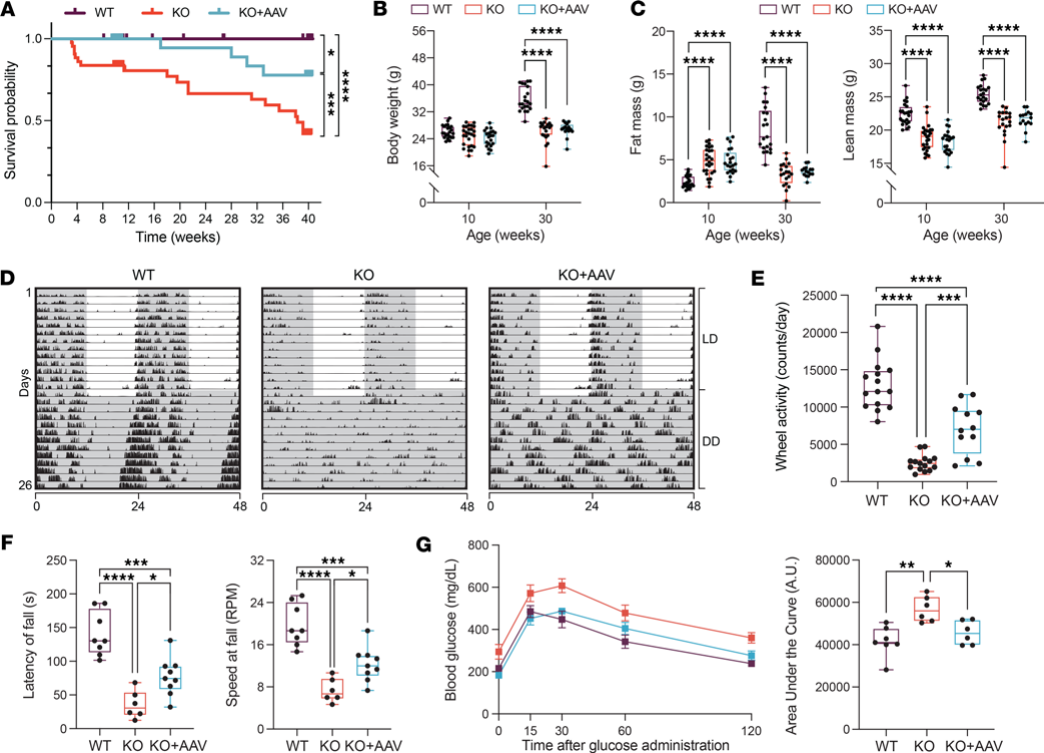
Muscle-specific rescue of *Bmal1* increases the survival rate of *Bmal1*-KO mice. (**A**) Kaplan-Meier survival curves of WT (*n* = 45), KO (*n* = 43), and KO+AAV (*n* = 36) mice. Log-rank test, *P < 0.05, ***P < 0.001, ****P < 0.0001. (**B**) Body weight measurements (*n* = 16–23 mice/group). Two-way ANOVA, ****P < 0.0001. (**C**) Body composition analysis (*n* = 15–23 mice/group). Two-way ANOVA, ****P < 0.0001. (**D**) Representative locomotor activity traces for individual mice (double plotted for visualization), showing 14 days in LD followed by 15 days in DD. (**E**) Voluntary wheel activity (*n* = 12–15 mice/group). One-way ANOVA, ***P < 0.001, ****P < 0.0001. (**F**) Latency to fall and running speed are increased in KO+AAV mice (*n* = 6–9 mice/group). One-way ANOVA, *P < 0.05, ***P < 0.001, ****P < 0.0001. (**G**) Glucose tolerance test and area under the curve (AUC) (*n* = 6–7 mice/group). One-way ANOVA, *P < 0.05, **P < 0.01. Box plots show the interquartile range, median (line), and minimum and maximum (whiskers).

*Bmal1*-KO mice have a median lifespan of approximately 37 weeks and are considered a model of premature aging (9). We investigated whether skeletal muscle–specific *Bmal1* rescue could increase their lifespan. We generated cohorts of male WT (*n* = 45), *Bmal1*-KO (*n* = 43), and *Bmal1*-KO+AAV (*n* = 36) mice and maintained them out to 40 weeks of age past the median lifespan of the *Bmal1*-KO in our facility (38.3 weeks). We found that the muscle-specific *Bmal1*-KO+AAV mice had an extended lifespan, with 88.9% (32/36) of them surviving through 40 weeks. In contrast, only 44.2% (19/43) of *Bmal1*-KO mice survived over the same period (Figure 2A). Using log-rank tests, we found that there was a statistically significant difference in survival between the *Bmal1*-KO and *Bmal1*-KO+AAV groups (*P* value < 0.0057). As expected, all WT mice survived (45/45; 100%), and there was a notable difference in survival between the WT mice and both the *Bmal1*-KO and *Bmal1*-KO+AAV mice, indicating that the AAV-mediated rescue of skeletal muscle *Bmal1* improves the lifespan of the *Bmal1*-KO mouse but that this is not sufficient to restore it completely to the WT status (Figure 2A).

One of the known phenotypes of the *Bmal1*-KO mice is that they exhibit limited increases in body weight after 10 weeks of age (9, 22). We tracked body weight weekly across all mice over 40 weeks (Supplemental Figure 2A). We found that at 10 weeks of age, there is no difference in the body weights of the WT, *Bmal1*-KO, and *Bmal1*-KO+AAV mice (Figure 2B). However, consistent with the literature, we observed that the *Bmal1*-KO mice stopped gaining weight at 12 weeks of age (9, 22). By approximately 30 weeks of age, the body weights of the *Bmal1*-KO as well as the KO+AAV mice were considerably lower than WT mice and were not different from each other. Thus, reexpressing *Bmal1* in skeletal muscle after birth was not sufficient to rescue the lack of postnatal growth that occurs in the *Bmal1*-KO mice (Supplemental Figure 2A).

Another phenotype of the *Bmal1*-KO mouse is that the distribution of lean and fat mass is different compared with the WT mice (22). We measured body composition from 10 to 40 weeks of age across the 3 groups, every 3 weeks (Supplemental Figure 2, B and C). We found that at 10 weeks, *Bmal1*-KO and KO+AAV mice had twice the amount of fat mass in grams (~109% higher) and ~17% lower lean mass than WT mice, while the body weights were the same across the 3 groups (Figure 2C). By ~30 weeks of age, when the KO and KO+AAV mice have lower body weights, the grams of fat mass declined dramatically to be ~60% lower in the KO and KO+AAV while lean mass remained lower by ~17% in WT mice (Figure 2C). Consistent with body weight, the expression of *Bmal1* in the muscle of the *Bmal1*-KO mice was not sufficient to alter the trajectory of body composition changes with age in the *Bmal1*-KO mouse.

We performed circadian and wheel activity phenotyping in these mice at 13 weeks of age. We kept the mice under normal 12-hour light/12-hour dark (LD) conditions for 2 weeks, followed by 2 weeks under constant conditions (total darkness, DD; the representative actograms from DD data are provided in Figure 2D). The WT mice exhibited clear rhythmic behavior under DD, with a normal period length of 23.74 hours (23). In contrast, both the *Bmal1*-KO and *Bmal1*-KO+AAV exhibited arrhythmic behavior in constant darkness, as expected (Figure 2D and Supplemental Figure 2D). We note that the *Bmal1*-KO and KO+AAV mice exhibited rhythmic cage activity behavior under LD conditions, and this has been previously shown (Supplemental Figure 2E) (24). We then analyzed the amount of voluntary wheel activity for the 3 different groups of mice for 7 consecutive days under LD conditions (*n* = 12–15 mice/group). We found that, consistent with McDearmon et al. (12), the muscle-specific expression of *Bmal1* was sufficient to markedly increase, by ~2.8-fold, the voluntary wheel activity when compared with the *Bmal1*-KO mice (Figure 2E). We also tested motor coordination and balance in a second cohort of 10-week-old mice using the rotarod test. With this assay, we found that the muscle-specific *Bmal1* expression improved balance, resulting in a longer time to fall (latency to fall) as well as a higher speed at fall compared with the *Bmal1*-KO mice (Figure 2F). For both wheel running and rotarod test measurements, the AAV+KO mice performed better than the *Bmal1*-KO mice but were not fully restored to WT levels. These outcomes verify that the muscle-specific expression of *Bmal1* in the KO mouse is sufficient to improve voluntary physical activity and neuromuscular parameters of mobility.

We performed glucose tolerance tests on a third cohort of 10-week-old mice at ZT4 (4 hours after lights on), as this is a metabolic parameter known to be disrupted in the *Bmal1*-KO mouse (10, 25). Consistent with previous findings, *Bmal1*-KO mice showed higher glucose peaks and slower clearance after an i.p. glucose injection (25). Muscle-specific *Bmal1* rescue reduced both the peak glucose rise and the time to return to baseline, resulting in a markedly lower area under the curve compared with *Bmal1*-KO mice, comparable to WT levels (Figure 2G). This suggests that *Bmal1* reexpression in skeletal muscle can restore glucose tolerance even when other tissue clocks are dysfunctional. We acknowledge there are temporal variations in the glucose tolerance test outcomes, as other groups have shown the importance of the liver and other tissues for systemic glucose homeostasis across the day (26). Despite not rescuing rhythmic behavior, the skeletal muscle–specific Bmal1 expression improved lifespan, cage activity, neuromuscular coordination, and systemic glucose metabolism.

### Muscle-specific expression of Bmal1 increases muscle-specific force but not fiber area or fiber type.

**Figure 3:**
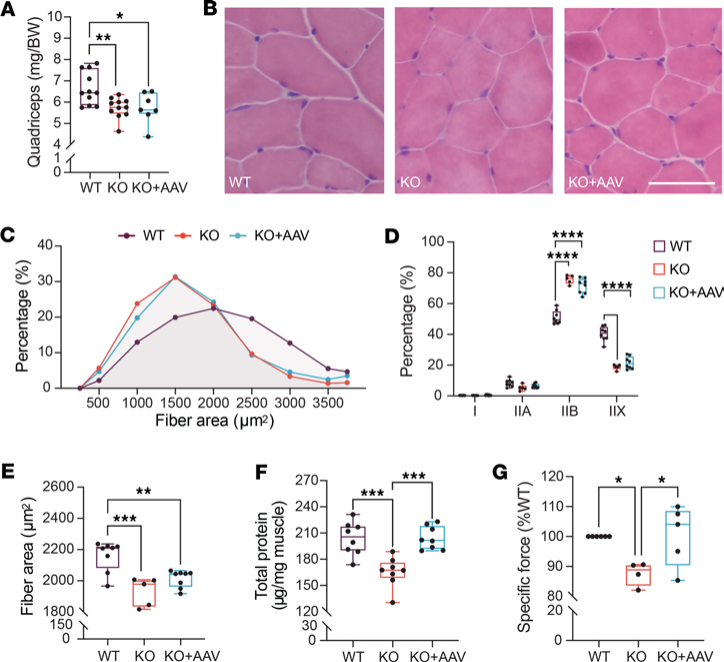
Functional and histological analysis from muscle-specific rescue of Bmal1 at 10 weeks of age. (**A**) Quadriceps weight normalized by body weight across groups (*n* = 7–11 mice/group). One-way ANOVA, *P < 0.05, **P < 0.01. (**B**) Representative images of hematoxylin and eosin staining from TA cross sections in WT, KO, and KO+AAV mice. Scale bar = 100 μm. (**C**) Muscle fiber cross-sectional area (CSA) in TA sections. (**D**) Muscle fiber type composition in TA cross sections (*n* = 5–8 mice/group). Two-way ANOVA, ****P < 0.0001. (**E**) Distribution of muscle fiber CSA (*n* = 5–8 mice/group). One-way ANOVA, **P < 0.01, ***P < 0.001. (**F**) Total protein concentrations (μg/mg muscle wet weight) in gastrocnemius muscle samples (*n* = 8 mice/group). One-way ANOVA, ***P < 0.001. (**G**) Extensor digitorum longus (EDL) specific force production in WT, *Bmal1*-KO, and KO+AAV mice (*n* = 4–6 mice/group). Measurements normalized to WT values. One-way ANOVA, *P < 0.05. Box plots show the interquartile range, median (line), and minimum and maximum (whiskers).

We next characterized the skeletal muscle phenotype from a cohort of 10-week-old mice to determine the cell- and tissue-specific effects of *Bmal1* in skeletal muscle. Consistent with our body weight and body composition analyses, the muscle weights from *Bmal1*-KO and *Bmal1*-KO+AAV were lower than WT and not different from each other (Figure 3A). Histological analysis of fiber size and fiber type (Figure 3, B–E) revealed no effects of AAV-*Bmal1* treatment on fiber size distribution or average fiber size as visualized by similar frequency distributions of both *Bmal1*-KO and KO+AAV mice compared with WT muscle. Fiber type analysis determined that *Bmal1* expression also did not result in a shift in the overall myosin heavy chain (MHC) isoform distribution (Figure 3D), as both KO and KO+AAV muscle exhibited similar fiber type distribution characterized by an increased number of MHCIIB-expressing fibers and a decrease in the number of IIX fibers, when compared with WT mice (Figure 3D). Deeper analysis of fiber area per fiber type showed that reexpression of *Bmal1* was sufficient to result in a small but notable increase in the average CSA of type IIA and type IIB fibers compared with KO, with no effect on type IIX fibers (Supplemental Figure 3). Thus, AAV reexpression of *Bmal1* in the *Bmal1*-KO mouse had no effect on muscle weight or fiber CSA, with only minor effects on fiber type, indicating that these muscle parameters do not contribute to the increased longevity of the KO+AAV mice.

Subjective viewing of the H&E stains of the *Bmal1*-KO muscle noted that the cytoplasm was, in general, paler in color compared with the WT muscle. We analyzed total protein content/muscle mass, as μg protein/mg muscle tissue, using a buffer system to solubilize all proteins in muscle from the 3 groups. Consistent with well-established measures of protein in mammalian skeletal muscle (27), we found that the protein content/mass was approximately 20.4% in WT muscle. Protein content/mass was markedly decreased in the *Bmal1*-KO muscle to approximately 16.5%, and reexpression of *Bmal1* in the muscle of the *Bmal1*-KO mouse was sufficient to restore protein content/mass to WT levels (Figure 3F). The restoration of protein concentration per muscle mass is important as it is estimated that over 80% of total protein in skeletal muscle is myofibrillar (e.g., myosin, titin). Thus, changes in total protein/mass reflect changes in many proteins that contribute to muscle mechanical function. We previously reported that skeletal muscle from *Bmal1*-KO exhibits reduced maximum isometric force normalized to physiological cross section, known as specific force (28). In this study, we performed ex vivo muscle mechanics on the EDL muscles of WT, *Bmal1*-KO, and *Bmal1*-KO+AAV mice at 10 weeks of age and found that the specific force of EDL muscles from *Bmal1*-KO mice was markedly reduced but that reexpression of *Bmal1* in the KO mice was sufficient to increase the specific force back to WT levels (Figure 3G). These results suggest that the improved muscle protein concentration in the AAV *Bmal1*-KO muscle was sufficient to support the rescue of muscle-specific force, and this functional improvement likely contributes to increased cage activity behavior and to improved neuromuscular parameters of mobility.

### Comprehensive multiomics reveals changes in substrate utilization in the Bmal1-KO+AAV mice.

**Figure 4:**
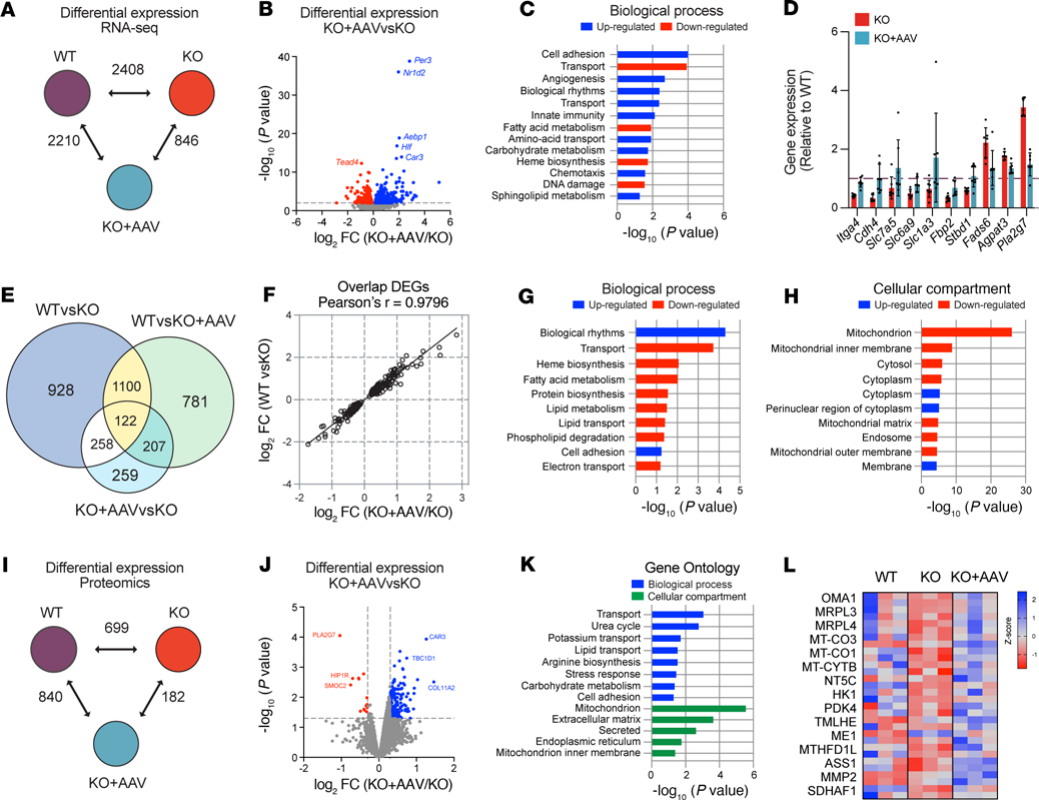
Multiomics analysis of gastrocnemius samples from 10-week-old rescue mice. (**A**) Number of differentially expressed genes (DEGs) in each comparison group. (**B**) Volcano plot showing *P* value and log_2_ fold-change of DEGs in RNA-Seq. Downregulated genes in red, upregulated in blue. (**C**) Biological processes enriched in DEGs. (**D**) Expression of genes associated with cell adhesion, amino acid transport, carbohydrate metabolism, and fatty acid metabolism from **C** (*n* = 7 samples/group). Expression is in counts per million, normalized to WT levels (dashed line). (**E**) Overlap analysis of DEGs from **A**. (**F**) Correlation of expression changes in 273 overlapping genes between WT versus KO and KO+AAV versus KO. (**G** and **H**) GO enrichment analysis for biological processes (**G**) and cellular compartments (**H**) enriched in overlapping genes. (**I**) Number of differentially expressed proteins (DEPs) in each comparison (*n* = 3 samples/group). (**J**) Volcano plot of DEPs in KO+AAV vs. KO. Downregulated proteins in red, upregulated in blue. (**K**) GO enrichment analysis for biological processes and cellular compartments of DEPs in KO+AAV vs. KO. (**L**) Heatmap analysis of proteins enriched for “Mitochondrion.” Each row represents ion intensity of a protein, with data *Z*-scaled. Blue indicates high abundance and red low abundance of proteins.

We conducted transcriptomics and proteomics on gastrocnemius muscles of 10-week-old WT, *Bmal1*-KO, and *Bmal1*-KO+AAV mice to identify potential molecular features associated with increased lifespan. At this age, body weights of *Bmal1*-KO and KO+AAV mice were similar to WT mice. RNA sequencing revealed 2,408, 2,210, and 846 differentially expressed genes (DEGs) in WT versus *Bmal1*-KO, WT versus KO+AAV, and KO+AAV versus KO groups, respectively (*P* < 0.01) (Figure 4A and Supplemental Data 1). The largest differences were between WT and both KO groups, with modest differences between *Bmal1*-KO versus KO+AAV. We focused on the 846 DEGs between KO+AAV versus KO, of which 574 genes were up- and 272 were downregulated, as presented in the volcano plot in Figure 4B. Notably, core clock genes *Per3* and *Nr1d2*, along with *Aebp1*, *Hlf*, and the muscle-specific carbonic anhydrase *Car3*, were upregulated by AAV-*Bmal1*, while the muscle-enriched transcription factor *Tead4* was markedly downregulated, suggesting reductions in the Hippo signaling pathway (Figure 4B). Before pathway analysis, we examined gastrocnemius from 10-week-old *Bmal1*-KO mice and *Bmal1*-KO mice systemically infected at postnatal day 5 with AAV9-CK6-GFP as a control for AAV transduction. We identified 66 overlapping DEGs between KO versus KO+GFP and KO versus KO+AAV (Bmal1) that were enriched for immune response genes and removed them from any further analysis (Supplemental Figure 4A).

Gene Ontology (GO) enrichment analyses were performed on DEGs to investigate biological processes altered in response to *Bmal1* expression in the *Bmal1*-KO muscle: a total of 13 biological functions were found to be significantly enriched (*P* value < 0.05) (Figure 4C and Supplemental Data 1). Upregulated processes included “Cell adhesion” with genes that contribute to cell surface adhesion and signaling, such as *Itga4* and *Cdh4* (Figure 4D). Two of the upregulated pathways in KO+AAV muscles were amino acid transport (e.g., *Slc7a5* or *Lat1*, *Slc6a9*, and *Slc1a3*) and carbohydrate metabolism (e.g., *Fbp2* and *Stbd1*) (Figure 4D). In the downregulated processes, we found that fatty acid metabolism was enriched in the *Bmal1*-KO+AAV, with genes such as *Pla2g7*, phospholipase A2 group VII, which regulates phospholipid catabolism during inflammatory and oxidative stress responses (Figure 4D). As seen in Figure 4D, fatty acid metabolism genes were highly expressed in muscles of *Bmal1*-KO compared with WT muscles. These results suggest that AAV-*Bmal1* regulated the expression of genes related to substrate metabolism in muscle, increasing the expression of genes related to carbohydrate and amino acid metabolism while decreasing the expression of those involved in lipid metabolism.

To evaluate the extent of the AAV-mediated rescue of *Bmal1* across different skeletal muscles, we measured the expression of some of the DEGs identified from the gastrocnemius muscle in TA, soleus, and quadriceps muscles from our model. We found that the gene expression changes in the TA and quadriceps muscles are similar to those seen in the gastrocnemius. In contrast, there was very little rescue of gene expression in the soleus muscles of the KO+AAV mice (Supplemental Figure 4C), consistent with the lack of AAV-CK6–driven expression of the *Bmal1* transgene in this muscle (Supplemental Figure 1A). To further evaluate the effects of the AAV-*Bmal1* treatment in muscles, we used changes in gene expression with loss of muscle *Bmal1* in the TA and soleus muscles reported by Dyar et al. (7) and asked if these clock output genes were modified in our AAV-*Bmal1* rescue model. We selected genes that lost circadian gene expression after knocking out *Bmal1* in the TA or the soleus muscle and that were differentially expressed at ZT0, close to our collection time ZT1. We found that the gene expression levels found in the TA identified genes in KO+AAV that responded to *Bmal1* reexpression and that were rescued to WT levels, while the expression analysis in the soleus identified genes that were not different in KO versus KO+AAV (Supplemental Figure 4D). These results verify that the AAV-*Bmal1* transduction in the *Bmal1*-KO mouse conferred changes in core clock and, importantly, in clock output gene expression across several different limb muscles. The lack of gene expression changes in the soleus muscle of our model is consistent with our results showing no detectable BMAL1-HA levels in the soleus and data from the literature showing limited transgenic expression driven by the CK6 promoter in the mouse soleus muscles.

We next hypothesized that the differences in the *Bmal1*-KO+AAV gene expression program would exhibit a set of genes that would be more similar to WT than KO muscle. Our first analysis was on the uniquely shared DEGs between the muscles of WT versus KO and WT versus KO+AAV. Out of 1,222 genes, we found that 1,100 DEGs were changing in the same direction in KO and KO+AAV when compared with WT, indicating that the expression of these genes was not rescued by AAV treatment (Venn diagram, Figure 4E). Consistent with our hypothesis, we identified 258 DEGs that were different between WT and KO muscle, as well as between KO versus KO+AAV, but were not DEGs in the WT versus KO+AAV comparison (Venn diagram, Figure 4E). In Figure 4F we plotted the log_2_ fold-change for each of the 258 genes, and we verified that the changes in the KO+AAV versus KO were similar in both magnitude and direction with those in the WT versus KO and exhibited a strong fold-change correlation (Pearson’s *r* = 0.9796, *P* < 0.0001) (Figure 4F). Functional analysis of these genes identified biological processes related to lipid metabolism that were enriched in the downregulated genes of the KO+AAV/WT groups, while biological rhythms, and cell adhesion were biological processes enriched in the upregulated genes (Figure 4G). Mitochondrion-related genes were downregulated in the KO+AAV in the same direction as the WT muscles (Figure 4H). Commonly upregulated genes included perinuclear region of cytoplasm, such as *Ntm*, *Cask*, *Mlf1*, *Ctif*, and *Stx4a*, which encode proteins located around the nucleus. Thus, while the impact of restoring *Bmal1* on gene expression in muscle is modest, there is a considerable cluster of both up- and downregulated genes that are restored to levels similar to WT.

### Proteomic analyses indicate that Bmal1 rescues the expression of mitochondrial localized proteins associated with the metabolism of carbohydrates and amino acids.

We used a tandem mass tag–based (TMT-based) quantitative proteomics approach with gastrocnemius samples (*n* = 3 samples/group) to define the protein changes in muscle with AAV treatment. Consistent with the RNA-Seq analysis, the proteomics data determined that the muscle of *Bmal1*-KO+AAV is more like *Bmal1*-KO muscle than WT. We identified differential expression of 699, 840, and 182 unique proteins in the WT versus *Bmal1*-KO, WT versus KO+AAV, and KO+AAV versus KO groups, respectively (Figure 4I). We focused on the 182 unique differentially expressed proteins; 169 proteins were upregulated in the KO+AAV group compared with the *Bmal1*-KO muscle, while only 13 proteins were downregulated as visualized in the volcano plot in Figure 4J. Initial inspection of the protein data identified that the upregulation of CAR3 and downregulation of PLA2G7 were consistent with the changes in the RNA-Seq data discussed above. We performed GO enrichment analysis to cluster the biological process and cellular components of the upregulated proteins (Figure 4K). The results indicated that carbohydrate metabolism (TBC1D1, PDK4, and HK1) and amino acid metabolism (urea cycle) pathways were upregulated, similar to the RNA-Seq results. The most considerable enrichment category for a cellular compartment was mitochondrion (Figure 4K). Examples of upregulated proteins located in the mitochondria are visualized in the heatmap in Figure 4L, which includes mitochondrial translation-related proteins (e.g., MRPL4, MRPL3), respiratory chain complex proteins (MT-CO1, MT-CO3), metabolism of carbohydrates (HK1 and PDK4), and metabolism of amino acids (e.g., MTHFD1L, ASS1, and MMP2). As noted above, mitochondrion was the most considerably enriched cellular compartment category for the downregulated DEGs from the KO+AAV RNA-Seq analysis (Figure 4H). Inspection of the RNA-Seq and proteomic gene lists found very few overlaps except for HIP1R, which was downregulated in both datasets, and NT5C and MTHFD1L, which were upregulated in both data sets. The rest of the mitochondrial genes/proteins were unique to each analysis. Therefore, our enrichment analysis of the proteomics results indicates that restoration of carbohydrate and amino acid metabolism and downregulation of fat metabolism are the primary outcomes of the AAV rescue of *Bmal1* in muscle at 10 weeks of age.

### Bmal1 reexpression restores genes associated with the mitochondria in the muscle of the 40-week-old Bmal1-KO mice.

**Figure 5:**
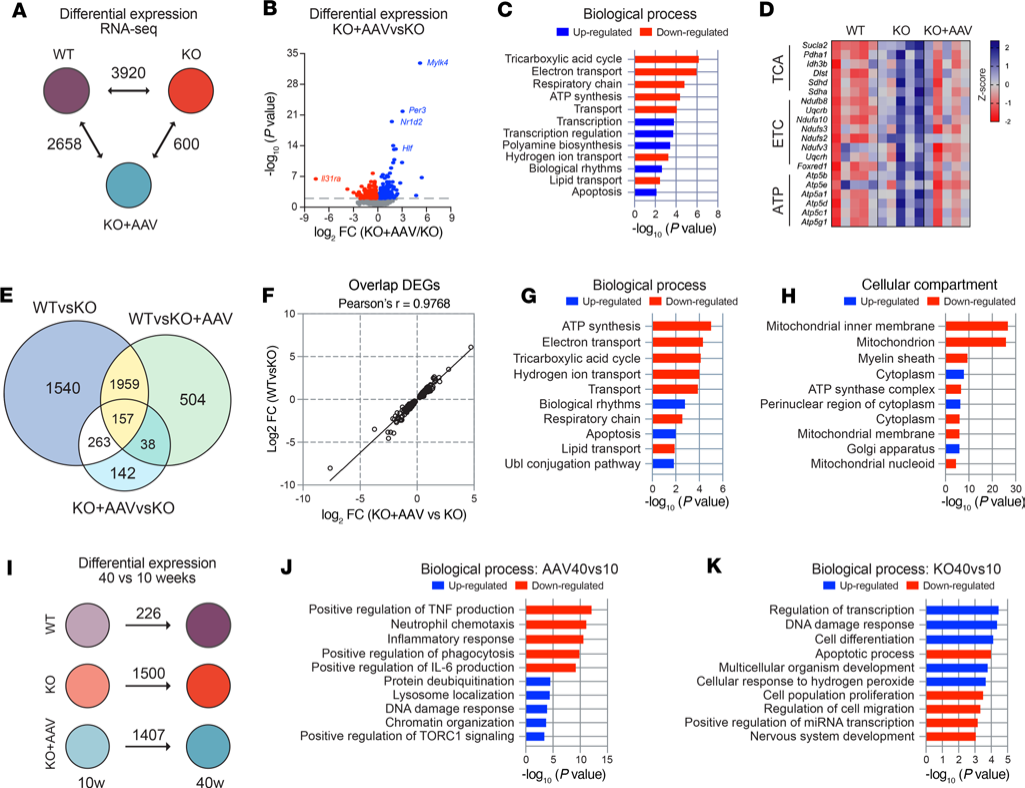
RNA-Seq analysis of gastrocnemius samples from 40-week-old rescue mice. (**A**) Number of DEGs in each comparison group (*n* = 5 samples/group). (**B**) Volcano plot showing *P* values and log_2_ fold-changes of DEGs in RNA-Seq. Downregulated genes are shown in red, upregulated in blue. (**C**) Biological processes enriched in DEGs. (**D**) Heatmap analysis of DEGs enriched for tricarboxylic acid, electron transport chain, and ATP synthesis from **C**. (**E**) Overlap analysis of DEGs from **A**. (**F**) Correlation of expression changes in 263 overlapping genes between WT vs. KO and KO+AAV vs. KO. (**G** and **H**) GO enrichment analysis for biological processes (**G**) and cellular compartments (**H**) enriched in overlapping genes. (**I**) Number of DEGs changing over time (40 vs. 10 weeks) in each group. (**J**) GO enrichment analysis for biological processes of DEGs down- or upregulated in KO+AAV. The top 10 overlapping processes are plotted based on –log_10_(*P* value). (**K**) GO enrichment analysis for biological processes of DEGs down- or upregulated in KO. The top 10 overlapping processes are plotted based on –log_10_(*P* value).

We next defined the changes in gene expression in the muscle of mice at 40 weeks of age, just beyond the median lifespan of the *Bmal1*-KO mice and at a time when both groups of mice are lower in body weight and have lost considerable fat mass compared with WT mice. The initial analysis determined that circadian clock gene expression was still engaged in the muscle at 40 weeks, consistent with the continued presence of the *Bmal1*-HA AAV. We identified that the changes in gene expression of the muscle of the KO compared with WT were even greater at 40 weeks of age (3,920 at 40 weeks vs. 2,408 at 10 weeks). However, the number of DEGs for WT vs. KO+AAV at this age was 2,658, which is only slightly higher than the 2,210 DEGs detected at 10 weeks, suggesting fewer changes in the KO+AAV muscle. Consistent with the 10-week analysis, the differences between the KO muscle versus KO+AAV were relatively small with a total of 600 DEGs (*P* value < 0.01) (Figure 5A and Supplemental Data 1). The volcano plot for the KO+AAV versus KO DEGs in Figure 5B indicated that the same numbers of genes were upregulated (*n* = 300) and downregulated (*n* = 300). Similar to our analysis of the 10-week-old mouse data, GO enrichment analysis revealed that mitochondria-related pathways were decreased in the KO+AAV muscles, and these include electron transport, tricarboxylic acid cycle, respiratory chain, and ATP synthesis (Figure 5C). A heatmap of genes from each of those categories is provided in Figure 5D. In general, continued *Bmal1* reexpression in the *Bmal1*-KO muscle functioned to bring these clusters of genes back to levels more similar to that seen in the WT muscle.

Overlapping analysis revealed that 1,959 DEGs were shared and changing in the same direction between WT versus KO and WT versus KO+AAV, indicating these genes were not rescued by AAV-*Bmal1* (Figure 5E). Consistent with our previous analysis, we identified 263 DEGs that were different between WT versus KO muscle, as well as between KO versus KO+AAV, but were not DEGs in the WT versus KO+AAV comparison (Figure 5E). These genes shared the same differential expression compared to KO, indicating these genes were rescued by AAV-*Bmal1* (Pearson’s *r* = 0.9768, *P* < 0.0001) (Figure 5F). Functional analysis of these genes identified that biological processes related to mitochondrion-related processes were enriched in the downregulated genes of the KO+AAV/WT groups, while biological rhythms and apoptosis are examples of biological processes enriched in the upregulated genes (Figure 5G). Mitochondrion-related genes were downregulated in the KO+AAV in the same direction as the WT muscles (Figure 5H), as previously shown in Figure 5D. Several studies have shown that loss of muscle *Bmal1* leads to impaired metabolic flexibility with a diminished ability to utilize glucose (6, 7). Our findings demonstrate that the use of AAV to restore *Bmal1* in the muscle of the global *Bmal1*-KO mouse supports improved glucose utilization and metabolic flexibility and rescues mitochondrial gene expression to WT levels.

### Muscle-specific expression of Bmal1 decreases the expression of genes associated with inflammation with aging in the muscles of the Bmal1-KO.

We next analyzed the changes in gene expression that occur with aging across each genotype by comparing gene expression from 10 to 40 weeks within each genotype to identify potential genes/pathways associated with increased longevity of the *Bmal1*-KO+AAV (Figure 5I). It was important to note that muscle gene expression did not change markedly in the WT mice, as only 226 genes were differentially expressed at 40 versus 10 weeks (*P* value < 0.01). In contrast, the muscle of *Bmal1*-KO and KO+AAV mice showed larger changes in gene expression likely reflecting the contributions of systemic factors to changes in muscle gene expression. The muscle of the *Bmal1*-KO mice exhibited 1,500 DEGs changing over time, while 1,407 DEGs were changing in the KO+AAVs (Figure 5I). We determined that approximately 35% of the DEGs with aging were in common between the KO and KO+AAV datasets, indicating that age-associated changes in gene expression were not largely shared (Supplemental Figure 5A). We looked at the top aging biological processes enriched within the KO+AAV, and we found that inflammation-related biological processes were downregulated in the muscles with age (Figure 5J). In contrast, the biological processes associated with aging-related pathways, such as DNA damage response or antioxidant response (cellular response to hydrogen peroxide), were upregulated in KO muscle (Figure 5K). We looked at the GO biological processes that were shared across both groups but were regulated in different directions (Supplemental Figure 5B and Supplemental Data 1). We identified 22 biological processes downregulated with age in the KO+AAV that were upregulated in the KO (e.g., Tumor necrosis factor production and Regulation of NF-kB transcription factor activity) and 4 biological processes linked to chromatin regulation and transcription that were upregulated in KO+AAV and downregulated in KO. The most considerably enriched category was positive response to TNF production, an important pro-inflammatory pathway (examples of genes with changes over time in expression are shown in Supplemental Figure 5B). Analysis of the gene expression changes with age highlights that the KO+AAV muscle was able to maintain metabolic pathways and that this was associated with reduced indicators of inflammation compared with the muscle of the aging *Bmal1*-KO mice.

### Bmal1 reexpression in skeletal muscle affects markers of systemic inflammation of Bmal1-KO mice.

**Figure 6:**
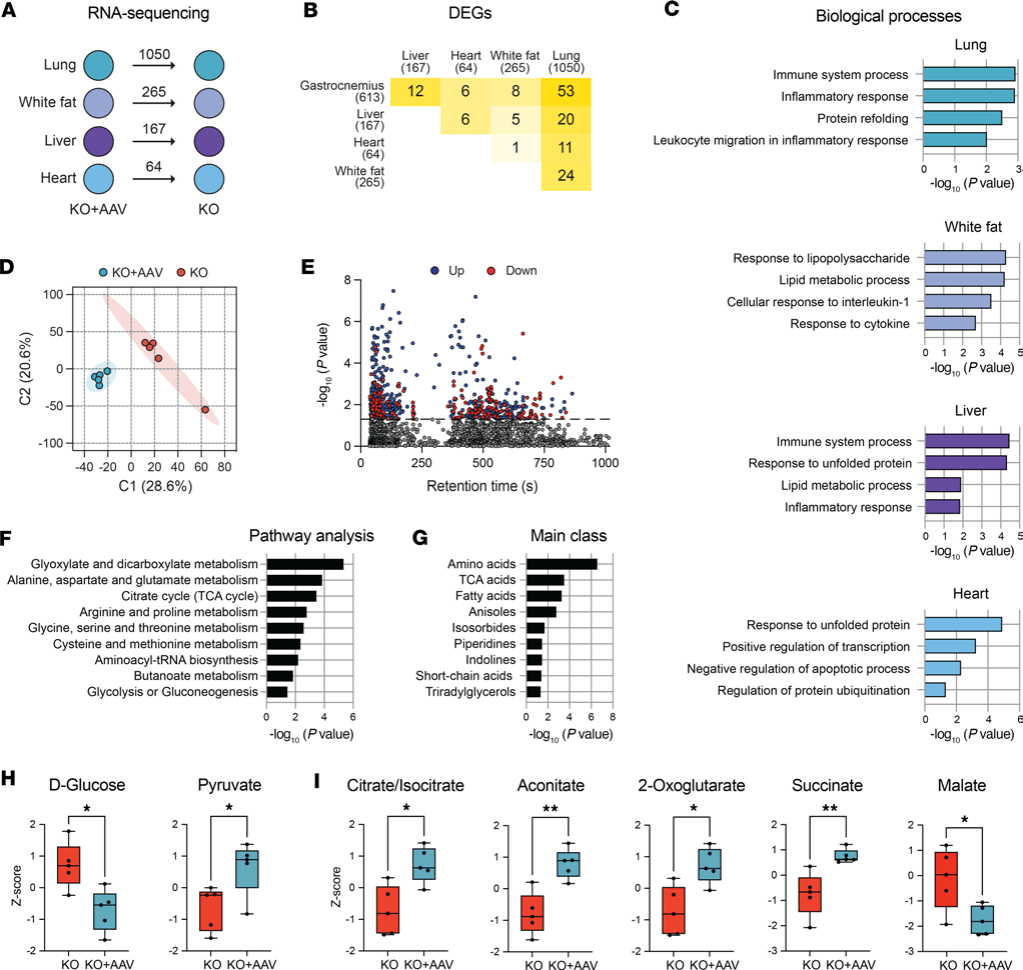
Systemic effects in the rescue model are associated with inflammatory status at 40 weeks of age. (**A**) Number of DEGs in liver, lung, heart, and white fat tissues comparing *Bmal1*-KO vs. KO+AAV at 40 weeks of age (*n* = 4 samples/group). (**B**) No overlapping DEGs were found across all tissues. (**C**) Examples of biological processes related to inflammation or metabolism in each tissue. (**D**) Partial least squares discriminant analysis of 4,129 metabolomic features. (**E**) Manhattan plot. The dotted line represents *P* value < 0.05 comparing *Bmal1*-KO with KO+AAV plasma samples (*n* = 5/group). Blue and red dots above the line indicate significantly increased or decreased features, respectively. (**F**) KEGG pathway analysis of untargeted plasma metabolomics results. (**G**) Main classes of plasma metabolites. (**H**) Metabolites related to alanine, aspartate, and glutamate metabolism. (**I**) Metabolites associated with inflammatory responses (*n* = 5 samples/group). Two-tailed Student’s, *P < 0.05, **P < 0.01. Box plots show the interquartile range, median (line), and minimum and maximum (whiskers).

To explore the systemic effects of the muscle-specific AAV rescue model, we performed RNA-sequencing of lung, heart, liver, and white adipose samples from 40-week-old *Bmal1*-KO and KO+AAV mice (*n* = 4 samples/group). Analysis of DEGs found that the lung exhibited the greatest effect with a total of 1,050 DEGs, followed by white fat, liver, and heart, showing 265, 167, and 64 DEGs, respectively (Figure 6A). DEGs showed tissue-specific effects of muscle rescue since very little overlap was found across all tissues (Figure 6B). We performed GO enrichment analysis for biological processes for each tissue dataset to address if any common pathways changed in these peripheral tissues of the *Bmal1*-restored mice and found very little overlap across tissues. However, we noted that the general categories of inflammation and substrate metabolism–related pathways were consistent in each of the tissues and that the stress response pathways were commonly regulated in the lung, liver, and heart tissues (Figure 6C). These findings support a model in which the rescue of more metabolic flexibility in the muscle of the *Bmal1*-KO+AAV is associated with the restoration of systemic metabolism and a concomitant reduction of systemic inflammation in the *Bmal1*-KO tissues.

To better understand the effects of the muscle-specific *Bmal1* expression on metabolic health, we performed untargeted metabolomics in plasma samples of both groups of 40-week-old mice (*n* = 4 samples/group). Plasma samples were collected at ZT1, the same time point as our RNA-Seq analyses. Following data extraction and filtering, 4,129 metabolite peaks were detected, and notable differences in the metabolite profiles were evident, with distinct separation of the groups (Figure 6D). In KO+AAV versus KO plasma samples, 435 metabolites increased and 224 decreased (*P* < 0.05) (Figure 6E). Mummichog analysis identified 551 empirical compounds linked to 399 Kyoto Encyclopedia of Genes and Genomes (KEGG) IDs.

We used the KEGG *Mus musculus* reference map for pathway enrichment, which identified alterations in amino acid metabolism, TCA cycle, butanoate metabolism, and glycolysis (Figure 6F). In addition, main class enrichment analysis identified that amino acids were the largest cluster of metabolites changing in the plasma after muscle-specific rescue of *Bmal1*, followed by TCA acids (Figure 6G). Consistent with our model, plasma glucose was decreased in the *Bmal1*-KO+AAV mice (Supplemental Figure 6A), and TCA intermediaries were increased in the plasma samples of *Bmal1*-KO+AAV compared with *Bmal1*-KO (Supplemental Figure 6B). The plasma levels of amino acids alanine, aspartate, glutamate, and lysine that can be metabolized to support the TCA cycle were increased in the *Bmal1*-KO+AAV (Figure 6H), suggesting the changes in carbohydrate metabolism in muscle support TCA intermediaries and likely decrease the use of plasma/systemic supplies of key amino acids. We note that these amino acids were rescued to levels closer to those seen in the WT mice (Supplemental Figure 6C). The 3 aromatic amino acids, l-phenylalanine, l-tyrosine, and l-tryptophan, were found to be decreased in the plasma samples of the *Bmal1*-KO+AAV, and this would be consistent with observations that they are associated with inflammatory status of multiple diseases (29–31) (Figure 6I). These results support our working model that rescuing the expression of *Bmal1* in the muscle of the *Bmal1*-KO affects the carbohydrate metabolism and amino acid needs to maintain the glycolysis-TCA cycle flux in the muscle of *Bmal1*-KO. Since muscle accounts for a large proportion of body mass, these metabolic effects across skeletal muscles have a broad metabolic and subsequent systemic immune impact across the organism that is associated with improvements in lifespan.

## Discussion

In this investigation, we found that the use of AAV to direct constitutive reexpression of the core clock factor, *Bmal1*, in skeletal muscle of the short-lifespan global *Bmal1*-KO mouse is sufficient to substantially extend survival. Combining mouse and muscle phenotyping at 2 ages with multiomics analyses of skeletal muscle, plasma, liver, white fat, heart, and lung, we propose that the primary effect of muscle *Bmal1* restoration is through rescuing skeletal muscle’s ability to utilize glucose with secondary changes to lipid and protein/amino acid metabolism. The ability to utilize glucose spares the need for amino acids to support the TCA cycle, which leads to restoring normal muscle protein concentration, with conserved measures of muscle strength (i.e., specific force) and marked improvements in activity behavior and mobility. Last, we suggest that the improvement in muscle metabolic flexibility has a substantial systemic impact through improvements in metabolism and markers of inflammation across several peripheral tissues. Thus, our analysis of the skeletal muscle–specific rescue of *Bmal1* in the *Bmal1*-KO mouse provides important new insights about the mechanisms through which *Bmal1* and reengagement of core clock and clock output genes in skeletal muscle support metabolic homeostasis and the consequent impact of muscle health on systemic homeostasis, inflammation, and lifespan (32–35).

Several epidemiological studies over the last 20 years have shown that maintenance of skeletal muscle strength is associated with lower mortality and morbidity rates with age as well as several chronic diseases (36–38). Preclinical studies have demonstrated that improving muscle function in a mouse model of cancer cachexia prolongs the overall survival of the mice (39). In Drosophila, muscle-specific activation of the pro-longevity transcription factor FoxO improves muscle function and extends lifespan (40). This study herein provides data that delineate molecular, metabolic, and phenotypic features of skeletal muscle health that contribute systemically to lifespan. Specifically, by restoring muscle *Bmal1* expression in the *Bmal1*-KO mouse, we reengage gene expression of core clock factors and several muscle-specific clock output genes. The improvement in glucose metabolism is consistent with several loss-of-function studies that put *Bmal1* and the core circadian clock mechanism as a critical upstream regulator of expression for several key glucose metabolism proteins, including HK1 and PDK4 (6, 7). Thus, we suggest that the outcomes from this study provide insight into potential mechanisms through which simple muscle health measurements can serve as a biomarker of both muscle and systemic health.

This study is not the first one to test the contribution of rescuing *Bmal1* in skeletal muscle. McDearmon et al. in 2006 used transgenic mice that constitutively expressed *Bmal1* under the control of human alpha-skeletal actin (*HSA*) promoter crossed with the global *Bmal1*-KO mice to test for tissue-specific restoration of rhythmic behavior (12). The HSA promoter is skeletal muscle specific, is expressed before birth in developing mouse muscle, and continues to be expressed through adulthood (41). The authors reported that the muscle-specific *Bmal1* rescue did not contribute to rhythmic behavior, but it resulted in increased voluntary wheel-running behavior. They also noted that by the end of their study (16–24 weeks) the muscle-specific *Bmal1* rescue mice were alive while the brain-specific *Bmal1* rescue mice were dying (12). Smith et al. used a newer *Bmal1*-stop-FL KO mouse model (13) crossed with the HSA-Cre mouse to restore endogenous *Bmal1* gene expression in skeletal muscle. They found improvements in glucose metabolism but survival effects were not reported (16). Most recently, Kumar et al. (42) used the same HSA-Cre to rescue endogenous *Bmal1* expression in a *Bmal1*-stop-FL KO mouse model, but they did not detect any improvements in muscle strength or lifespan. The different muscle function and survival outcomes between our and these studies are intriguing. Our findings are closely aligned with the results from McDearmon et al. in 2006 and are based on restoring expression of *Bmal1* in skeletal muscle from either a transgene or AAV vector but not through the endogenous *Bmal1* gene. This suggests that restoring *Bmal1* via the endogenous *Bmal1* gene in the muscle of *Bmal1*-KO mice is not sufficient to improve lifespan. In contrast, the use of an introduced transgene or AAV vector that expresses *Bmal1* from a constitutive muscle-specific promoter (HSA or CK6) is sufficient to restore key clock output genes with downstream outcomes that affect muscle strength and survival. Future studies will be required to determine the molecular mechanisms that contribute to these strikingly different outcomes between models. Moreover, our results suggest the potential of using the exogenous constitutive expression of *Bmal1* in muscle as a potential antiaging intervention.

Our initial multiomics analyses focused on the samples from the 10-week-old mice because at this age there is no difference in total body weight among the groups of mice. Analysis of our multiomics data provided evidence that the major pathway affected in the *Bmal1*-rescued muscle was the increased ability to use glucose as substrate. This is consistent with previous work by our lab and others (6, 7, 16) that muscle fiber–specific loss of *Bmal1* leads to decreased glucose uptake and marked reductions of several enzymes within the glycolytic pathway (6). In addition, loss of muscle *Bmal1* was associated with increased gene expression to support fat metabolism and large increases in the free pool of many amino acids (6). The results of this study verify that the gain of *Bmal1* function in *Bmal1*-KO muscle was sufficient to improve pathways regulating glucose metabolism in muscle. This suggests that *Bmal1* and potentially the circadian clock are fundamental for the maintenance of glucose metabolism and metabolic flexibility. However, we acknowledge that a limitation of our study is that we did not directly measure metabolic flux in the skeletal muscles of these mice. Future experiments are needed to directly assess substrate metabolism and metabolic flux following the reexpression of *Bmal1* in the *Bmal1*-KO muscle. Last, we found that restoration of glucose metabolic pathways in skeletal muscle was sufficient to rescue systemic glucose tolerance outcomes at ZT4 in the *Bmal1*-KO mice, highlighting the substantial contribution of skeletal muscle to overall glucose maintenance. These effects were maintained through 40 weeks of age and provide further support for the important function of muscle *Bmal1* in metabolic flexibility and systemic health.

Prior studies have used genetic approaches to rescue *Bmal1* expression in the brain, liver, or epidermal tissues of the *Bmal1*-KO mouse but have not seen an extension of lifespan (13–15). Since the muscle-specific *Bmal1* rescue was sufficient to extend *Bmal1*-KO lifespan, we analyzed peripheral tissues and plasma to identify potential systemic pathways improved with the muscle *Bmal1* rescue. Transcriptome analysis of liver, lung, and white fat revealed that inflammatory pathways were the primary systemic feature that was improved in the muscle rescue mice. This was most strongly detected in the lung, and this link between healthy muscle and the lung has not previously been defined to our knowledge; however, studies highlighting improvement of lung inflammation with exercise are consistent with our findings (43, 44). Changes in inflammatory pathways were also enriched in white fat and liver, and previous studies have shown that maintenance of muscle glucose homeostasis helps prevent chronic inflammation (45, 46).

In conclusion, our investigation emphasizes the importance of maintaining skeletal muscle glucose metabolism and metabolic flexibility for muscle function, mobility, and lifespan. This study demonstrated that targeted reexpression of *Bmal1* engages the muscle clock and that this is critical for restoring glucose metabolic pathways with concomitant improvements in markers of metabolic flexibility. Importantly, this was sufficient to improve muscle strength and mobility and to restore glucose tolerance while reducing systemic inflammation and extending lifespan. These findings therefore underscore the critical role of the circadian clock in skeletal muscle function and metabolism and highlight the key role of skeletal muscle in systemic health and aging.

## Methods

### Sex as a biological variable.

Our study focused exclusively on male mice, so it is unclear whether the findings are fully applicable to female mice; further studies would be required to assess potential sex-specific differences.

### Vector design and AAV production.

Mouse *Bmal1* cDNA was subcloned into pAAV-CK6 with a C-terminus HA tag and confirmed by Sanger sequencing. AAV serotype 9 was produced using transient transfection of HEK293T cells (ATCC, CRL-3216) and CsCl sedimentation by the University of Massachusetts Medical School Viral Vector Core as previously described (47). Vector preparations were determined by droplet digital PCR, and purity was assessed by SDS-PAGE and silver staining.

### Animal use and vector delivery.

Heterozygous *Bmal1*-KO mice (strain 009100) were obtained from The Jackson Laboratory. Mice were housed at the University of Florida animal care servicesfacility on a 12-hour light/12-hour dark cycle with free access to food and water. *Bmal1*-KO mice were bred from heterozygous parents, with genotype and sex determined as previously described (24, 48). Neonatal mice were genotyped on day 3, and on day 5, male homozygotes were cryoanesthetized and injected subxiphoidally with 20 μL containing 2 × 10^11^ genome copies of AAV as previously described (49). Pups were rewarmed and returned to their cages. WT controls were littermates.

### Survival study.

Mice were monitored daily for signs of illness and deaths were recorded. Animals that were close to dying were euthanized using CO_2_ asphyxiation and recorded. The decision to euthanize them was made by a veterinarian according to Association for Assessment and Accreditation of Laboratory Animals guidelines. Animals that were euthanized were considered censored deaths.

### Western blotting.

Proteins were isolated from gastrocnemius, diaphragm, TA, quadriceps, soleus, stomach, heart, lung, and liver tissues using RIPA buffer. For HA detection, 10 μg of protein was loaded per lane and 50 μg for BMAL1. Membranes were stained with Rev700 to assess total protein. Primary antibodies against HA (1:2,000; Roche 11867423001) or BMAL1 (1:1,000; Cell Signaling Technology 14020) were incubated overnight. Blots were probed with HRP-conjugated anti-rat (1:5,000; Invitrogen 31470) for HA or anti-rabbit (1:10,000; Invitrogen A24531) for BMAL1. Detection was done with Pierce ECL Western Blotting Substrate (Thermo Fisher Scientific) and imaged using a Chemidoc system (Bio-Rad).

### ChIP assay.

One whole frozen quadriceps was minced, then sonicated (Omni GLH homogenizer) in buffer (10 mM of HEPES, pH 7.5, 10 mM MgCl_2_, 60 mM KCl, 300 mM sucrose, 0.1 mM EDTA, pH 8.0, 0.1% Triton X-100, 1 mM DTT, 1 mM PMSF, and protease inhibitors) containing 1% formaldehyde. After 15 minutes, crosslinking was stopped with glycine, and tissue was centrifuged 300*g* for 5 minutes at 4°C. The pellet was resuspended in 0.5× RIPA buffer with protease inhibitors, 1 mM PMSF, and 1 mM DTT. Nuclei were released via sonication, and 10 μL of genomic DNA was taken as input. Samples were incubated overnight with anti-BMAL1 antibody (3 μg, MilliporeSigma, SAB4300614) and Dynabeads protein A/G for 4 hours at 4°C. Beads were washed with RIPA and Tris-EDTA buffers. DNA/antibody complexes were eluted, then incubated with proteinase K. DNA was purified using the QIAGEN PCR kit and resuspended in TE buffer. qPCR was performed on 2 μL of DNA, with enrichment normalized to input DNA. ChIP experiments were performed with independent chromatin preparations, and qPCRs were done in duplicate. Primer details are in Supplemental Table 1.

### Diurnal behavior and wheel activity levels.

Mice were individually housed on a 12-hour light/12-hour dark schedule, and wheel-running activity was recorded for 2 weeks, followed by 2 weeks in constant darkness using the ClockLab system (Actimetrics). Wheel-running activity from 7 consecutive days per mouse was analyzed using ClockLab software for statistical analysis.

### Motor coordination.

Motor coordination was assessed with the rotarod test (Rotamex 5, Columbus Instruments) with rod acceleration from 4 to 40 rpm over 5 minutes. Three trials were conducted on the testing day, with 5-minute rests in between. The average of the last 3 trials was used to determine the final latency to fall and speed scores.

### Body composition analysis.

Body weight and body composition were recorded at 3-week intervals starting at 10 weeks of age in *Bmal1*-KO (*n* = 12–26), KO+AAV (*n* = 14–21), and WT (*n* = 19–23) mice. Body composition was assessed by nuclear magnetic resonance using an EchoMRI whole body composition analyzer (EchoMedical Systems).

### RNA extraction.

Total RNA was isolated from gastrocnemius, heart, liver, lung, and white fat tissues using TRIzol reagent (Invitrogen) and QIAGEN RNeasy Mini Kit. Frozen tissues were homogenized in TRIzol with 1.0 mm zirconium oxide beads using a Bullet Blender at 4°C. After a 5-minute incubation, homogenates were mixed with chloroform and centrifuged at 8,000*g* for 15 minutes at 4°C. The aqueous phase was mixed with isopropanol, transferred to an RNeasy Mini spin column, and treated with RNase-free DNase (QIAGEN). RNA was eluted in nuclease-free water.

### qPCR.

A total of 1,000 ng of total RNA from gastrocnemius, soleus, TA, and quadriceps was reverse-transcribed using SuperScript VILO Master Mix (Invitrogen). A total of 20 ng of cDNA was used as template. Gene expression was measured in duplicate with Fast SYBR Green Master Mix (Thermo Fisher Scientific) and 400 nM primers in a 20 μL reaction volume on a QuantiStudio 3 thermocycler (Applied Biosystems). Relative expression was calculated using the 2^ΔΔCT^ method and *Gapdh* as reference gene. Primer sequences are listed in Supplemental Table 2.

### RNA-sequencing and bioinformatics analysis.

Purified RNA samples had an RNA integrity number above 8.0 and were sequenced at the University of Florida’s Interdisciplinary Center for Biotechnology Research (ICBR) and Novogene (UC Davis). Poly(A)-selected RNA-Seq libraries were prepared with 250 ng of RNA using the Illumina mRNA Prep kit. Libraries from heart, lung, white fat, and liver, and 4 replicates of 10-week-old gastrocnemius were sequenced on ICBR’s Illumina NovaSeq 6000 (2 × 150 bp) to a minimum of 40,000 reads per sample. Libraries from the remaining gastrocnemius samples were sequenced on Novogene’s Illumina NovaSeq 6000 (2 × 150 bp) to achieve at least 20,000 reads per sample. FastQ files were downloaded to the University of Florida HiPerGator computing cluster. FastQ files were processed on the HiPerGator cluster. Reads were aligned to the *Mus musculus* genome GRCm38 (mm10) using HISAT2 v2.2.1 and annotated with HTseq-counts. Differential expression was analyzed with DESeq2, using sequencing site as a covariate, and filtered for genes with mean DESeq2 normalized counts > 5. GO enrichment was analyzed using Database for Annotation, Visualization and Integrated Discovery v2022q1 with *P* < 0.01. Raw counts are included in Supplemental Data 1.

### Immunohistochemistry.

TA muscle from 10-week-old mice was embedded in OCT compound (Tissue-Tek, Milles Inc.), frozen in 2-methylbutane (Thermo Fisher Scientific, O3551-4) cooled in liquid nitrogen, and stored at –80°C. Sections (10 μm thick) were cut with a Microm HM 505 E cryostat. For BMAL1 detection, sections were fixed in 4% paraformaldehyde, permeabilized with 0.5% Triton X-100, and blocked with 5% BSA, 10% normal goat serum, 1% glycine, and 0.1% Triton X-100 in PBS for 1 hour at room temperature. Primary antibodies (rabbit anti-BMAL1, 1:500; Novus Biologicals [Bio-Techne] NB100-2288, and mouse anti-Dystrophin conjugated with Alexa Fluor 488 [1:50; Santa Cruz Biotechnology sc-73592]), were incubated overnight at 4°C. Sections were then incubated with goat anti-rabbit Alexa Fluor 568 and counterstained with DAPI (1:10,000; Invitrogen D1306). For fiber typing, unfixed sections were incubated overnight with primary antibodies from the Developmental Studies Hybridoma Bank: IgG2b anti-MYH7 (Type 1, 1:100, BA.D5), IgG1 anti-MYH2 (Type 2A, neet, SC.71), IgM anti-MYH4 (Type 2B, neet, BF.F3), and rabbit anti-laminin (1:100; MilliporeSigma L9393). Sections were then incubated with Invitrogen’s secondary antibodies (IgG1 Alexa Fluor 647, 1:250; IgG2b Alexa Fluor 488, 1:500; IgM Alexa Fluor 594, 1:250; goat anti-rabbit IgG Alexa Fluor 405, 1:100), washed, and postfixed in methanol. Sections were mounted in ProLong antifade medium. Images were captured with a Leica DMi8 microscope (20× objective). CSA assessment and fiber typing analysis were performed using MyoVision 2.0, as previously reported (50).

### AAV vector genome detection.

AAV vector copy numbers were quantified in gastrocnemius, quadriceps, soleus, tibialis anterior, heart, and liver tissues from KO+AAV mice (*n* = 5–7). Genomic DNA was isolated using DNeasy Blood & Tissue kit (QIAGEN). qPCR was performed using a QuantiStudio 3 thermocycler (Applied Biosystems) with an inverted terminal repeat-specific primer set (Fwd: 5′-GGAACCCCTAGTGATGGAGTT-3′; Rev: 5′-CGGCCTCAGTGAGCGA-3′) (51) and Fast SYBR Green Master Mix (Thermo Fisher Scientific). A standard curve was generated with serial dilutions, 10^2^–10^8^, of AAV2-CK6-Bmal1-HA plasmid. Results were expressed as mean AAV vector copy number per microgram of genomic DNA.

### Ex vivo muscle function.

Maximal tetanic tension of the EDL muscle from WT (*n* = 6), *Bmal1*-KO (*n* = 4), and *Bmal1*-KO+AAV (*n* = 5) was assessed by the University of Florida Physiological Assessment Core, as previously described (52). Specific tension and physiological CSA were calculated using standard equations (53), with data normalized to WT values for comparison.

### Whole muscle protein quantification.

Protein was isolated as described by Roberts et al. (27). Gastrocnemius muscle from 8 mice per group was weighed and placed in ice-cold buffer (25 mM Tris, pH 7.2, 0.5% Triton X-100, proteinase inhibitor) at 20× volume. Muscles were homogenized with glass Dounce grinders. Protein concentration was measured using the Microplate BCA protein assay kit (Thermo Fisher Scientific) and expressed per milligram of wet tissue.

### LC-MS untargeted plasma metabolomics.

Plasma samples (*n* = 5; 4 per group plus 1 pooled) were processed by the University of Florida Southeast Center for Integrated Metabolomics. Samples were prenormalized by protein content. Global metabolomics profiling was conducted using a Thermo Fisher Scientific Q-Exactive Orbitrap mass spectrometer with Dionex UHPLC, as reported before (54). Extracts were spiked with 1 μL of a standard mixture for targeted carnitine identification. Samples were analyzed in positive and negative heated electrospray ionization with 35,000 mass resolution at *m/z* 200. Separation was performed on an ACE 18-pfp column using 0.1% formic acid (A) and acetonitrile (B). The flow rate was 350 μL/min with a column temperature of 25°C. Injection volumes were 4 μL for negative and 2 μL for positive ions.

MZmine was used to identify, deisotope, align features, and fill gaps. Adducts and complexes were removed. Data from positive and negative ion modes were combined, yielding 1,420 features in the positive mode and 2,709 in the negative mode. Data were normalized to the sum of metabolites for each sample, log_2_-transformed, and autoscaled. Statistical and KEGG enrichment analyses were done with MetaboAnalyst v5.0. Mummichog analysis associated *m/z* ratios with possible metabolites.

### TMT-based proteome profiling.

Frozen gastrocnemius muscles were ground into powder with a liquid nitrogen–cooled mortar and pestle. The powder was homogenized in lysis buffer (50 mM HEPES, pH 8.5, 8 M urea, 0.5% sodium deoxycholate) at 4°C, then centrifuged at 11,200*g* for 10 minutes. Protein concentrations of supernatants were determined using Coomassie-stained gels with BSA as a standard (55). For TMT labeling, 100 μg of each sample was digested with LysC (Wako) at 1:100 (w/w) for 2 hours, followed by Trypsin (Promega) at 1:50 (w/w) for at least 3 hours. Peptides were reduced with 1 mM DTT, alkylated with 10 mM iodoacetamide, and quenched with 30 mM DTT. Samples were acidified with trifluoroacetic acid, desalted using C18 cartridges, and dried. The peptides were resuspended in 50 mM HEPES (pH 8.5) and labeled with 10-plex TMT reagents. Labeled samples were mixed, desalted, and fractionated using basic pH reverse-phase chromatography (Agilent 1220). Fractions were analyzed by acidic pH reverse-phase LC-MS/MS on an Orbitrap Fusion mass spectrometer (Thermo Fisher Scientific) with data-dependent acquisition.

MS/MS raw files were processed using JUMP. Data were searched against the UniProt mouse database with a reversed decoy database. Search parameters included 15 ppm mass tolerance for precursor and fragment ions, fully tryptic restriction, and static modifications for TMT tags and carbamidomethylation, with dynamic modification for Met oxidation. Proteins were quantified by summing reporter ion counts across matched eptide-spectrum matches. Statistical analysis used a *P* < 0.05 and a fold-change threshold of ±0.3 log_2_FC, as reported before (56). Results are available in Supplemental Data 2.

### Statistics.

Data are presented as mean and SD or SEM. The unpaired 2-tailed Student’s *t* test was used for comparing 2 normally distributed groups. For comparing 3 groups, 1-way ANOVA with Tukey’s multiple-comparison post hoc test was applied for qPCR results, ChIP-qPCR, wheel activity, rotarod, AUC, or muscle weight. Ordinary 2-way ANOVA with Tukey’s multiple-comparison post hoc test was used for body weight or body composition over time, considering time and group as main effects. The cumulative survival analysis was carried out using the Kaplan-Meier method and the log-rank test for comparing survival curves. The Gehan-Breslow-Wilcoxon test was also used to assess early death events, but only log-rank test *P* values are reported. A *P* value less than 0.05 was considered significant. All analyses were performed using GraphPad Prism software version 9.

### Study approval.

Procedures followed institutional guidelines approved by the University of Florida IACUC (Protocol 0018).

### Data availability.

RNA-Seq datasets generated in this study have been deposited in NCBI GEO (GSE232957). Metabolomics data are available through the NIH Metabolomics Workbench (PR001677). Proteomics data have been deposited in the ProteomeXchange Consortium via PRIDE (PXD054406). All data points shown in the graphs are provided in the Supporting Data Values file.

## Author contributions

MAGM and KAE designed research; MAGM, EER, CMD, MRV, VP, JP, LCH, and FD conducted experiments; MAGM and CAW acquired data; MAGM, CAW, CMD, HD, LCH, and ZH analyzed data; and MAGM and KAE wrote the manuscript.

## Supplementary Material

Supplemental data

Supplemental data set 1

Supplemental data set 2

Unedited blot and gel images

Supporting data values

## Version 1 10/01/2024

In-Press Preview

## Version 2 11/22/2024

Electronic publication

## References

[1] Cox KH, Takahashi JS (2019). Circadian clock genes and the transcriptional architecture of the clock mechanism. J Mol Endocrinol.

[2] Gutierrez-Monreal MA (2020). Ticking for metabolic health: the skeletal-muscle clocks. Obesity (Silver Spring).

[3] Martin RA (2023). Metabolism and exercise: the skeletal muscle clock takes centre stage. Nat Rev Endocrinol.

[4] Guillaumond F (2005). Differential control of Bmal1 circadian transcription by REV-ERB and ROR nuclear receptors. J Biol Rhythms.

[5] Wolff CA (2023). Defining the age-dependent and tissue-specific circadian transcriptome in male mice. Cell Rep.

[6] Harfmann BD (2016). Muscle-specific loss of Bmal1 leads to disrupted tissue glucose metabolism and systemic glucose homeostasis. Skeletal Muscle.

[7] Dyar KA (2014). Muscle insulin sensitivity and glucose metabolism are controlled by the intrinsic muscle clock. Mol Metab.

[8] Andrews JL (2010). CLOCK and BMAL1 regulate MyoD and are necessary for maintenance of skeletal muscle phenotype and function. Proc Natl Acad Sci U S A.

[9] Kondratov RV (2006). Early aging and age-related pathologies in mice deficient in BMAL1, the core componentof the circadian clock. Genes Dev.

[10] Rudic RD (2004). BMAL1 and CLOCK, two essential components of the circadian clock, are involved in glucose homeostasis. PLoS Biol.

[11] Lefta M (2012). Development of dilated cardiomyopathy in Bmal1-deficient mice. Am J Physiol Heart Circ Physiol.

[12] McDearmon EL (2006). Dissecting the functions of the mammalian clock protein BMAL1 by tissue-specific rescue in mice. Science.

[13] Koronowski KB (2019). Defining the independence of the liver circadian clock. Cell.

[14] Welz P-S (2019). BMAL1-driven tissue clocks respond independently to light to maintain homeostasis. Cell.

[15] Petrus P (2022). The central clock suffices to drive the majority of circulatory metabolic rhythms. Sci Adv.

[16] Smith JG (2023). Liver and muscle circadian clocks cooperate to support glucose tolerance in mice. Cell Rep.

[17] Salva MZ (2007). Design of tissue-specific regulatory cassettes for high-level rAAV-mediated expression in skeletal and cardiac muscle. Mol Ther.

[18] Bartoli M (2006). Safety and efficacy of AAV-mediated calpain 3 gene transfer in a mouse model of limb-girdle muscular dystrophy type 2A. Mol Ther.

[19] Gregorevic P (2004). Systemic delivery of genes to striated muscles using adeno-associated viral vectors. Nat Med.

[20] Hauser MA (2000). Analysis of muscle creatine kinase regulatory elements in recombinant adenoviral vectors. Mol Ther.

[21] Gabriel BM (2021). Disrupted circadian oscillations in type 2 diabetes are linked to altered rhythmic mitochondrial metabolism in skeletal muscle. Sci Adv.

[22] Kettner NM (2015). Circadian dysfunction induces leptin resistance in mice. Cell Metab.

[23] Schwartz WJ, Zimmerman P (1990). Circadian timekeeping in BALB/c and C57BL/6 inbred mouse strains. J Neurosci.

[24] Bunger MK (2000). Mop3 is an essential component of the master circadian pacemaker in mammals. Cell.

[25] Kennaway DJ (2013). Global loss of bmal1 expression alters adipose tissue hormones, gene expression and glucose metabolism. PLoS One.

[26] Zhong L-X (2019). Circadian misalignment alters insulin sensitivity during the light phase and shifts glucose tolerance rhythms in female mice. PLoS One.

[27] Roberts MD (2020). An optimized procedure for isolation of rodent and human skeletal muscle sarcoplasmic and myofibrillar proteins. J Biol Methods.

[28] Schroder EA (2015). Intrinsic muscle clock is necessary for musculoskeletal health. J Physiol.

[29] Nikolaus S (2017). Increased tryptophan metabolism is associated with activity of inflammatory bowel diseases. Gastroenterology.

[30] Xu J (2020). Increased mortality of acute respiratory distress syndrome was associated with high levels of plasma phenylalanine. Respir Res.

[31] Capuron L (2011). Chronic low-grade inflammation in elderly persons is associated with altered tryptophan and tyrosine metabolism: role in neuropsychiatric symptoms. Biol Psychiatry.

[32] Gates AC (2007). Respiratory uncoupling in skeletal muscle delays death and diminishes age-related disease. Cell Metab.

[33] Vrailas-Mortimer A (2011). A muscle-specific p38 MAPK/Mef2/MnSOD pathway regulates stress, motor function, and life span in Drosophila. Dev Cell.

[34] Demontis F (2014). Intertissue control of the nucleolus via a myokine-dependent longevity pathway. Cell Rep.

[35] Demontis F (2013). The influence of skeletal muscle on systemic aging and lifespan. Aging Cell.

[36] Ruiz JR (2008). Association between muscular strength and mortality in men: prospective cohort study. BMJ.

[37] Landi F (2012). Sarcopenia as a risk factor for falls in elderly individuals: results from the ilSIRENTE study. Clin Nutr.

[38] Ling CHY (2010). Handgrip strength and mortality in the oldest old population: the Leiden 85-plus study. CMAJ.

[39] Zhou X (2010). Reversal of cancer cachexia and muscle wasting by ActRIIB antagonism leads to prolonged survival. Cell.

[40] Demontis F, Perrimon N (2010). FOXO/4E-BP signaling in Drosophila muscles regulates organism-wide proteostasis during aging. Cell.

[41] Brennan KJ, Hardeman EC (1993). Quantitative analysis of the human alpha-skeletal actin gene in transgenic mice. J Biol Chem.

[42] Kumar A (2024). Brain-muscle communication prevents muscle aging by maintaining daily physiology. Science.

[43] El-Mafarjeh E (2020). Exercise improves lung inflammation, but not lung remodeling and mechanics in a model of bleomycin-induced lung fibrosis. Oxid Med Cell Longev.

[44] Du S-F (2017). In obese mice, exercise training increases 11β-HSD1 expression, contributing to glucocorticoid activation and suppression of pulmonary inflammation. J Appl Physiol(1985).

[45] Shoelson SE (2006). Inflammation and insulin resistance. J Clin Invest.

[46] DeFronzo RA, Tripathy D (2009). Skeletal muscle insulin resistance is the primary defect in type 2 diabetes. Diabetes Care.

[47] Sena-Esteves M, Gao G (2020). Introducing genes into mammalian cells: viral vectors. Cold Spring Harb Protoc.

[48] Tunster SJ (2017). Genetic sex determination of mice by simplex PCR. Biol Sex Differ.

[49] Bish LT (2008). Adeno-associated virus (AAV) serotype 9 provides global cardiac gene transfer superior to AAV1, AAV6, AAV7, and AAV8 in the mouse and rat. Hum Gene Ther.

[50] Viggars MR (2022). Automated cross-sectional analysis of trained, severely atrophied, and recovering rat skeletal muscles using MyoVision 2.0. J Appl Physiol (1985).

[51] Aurnhammer C (2012). Universal real-time PCR for the detection and quantification of adeno-associated virus serotype 2-derived inverted terminal repeat sequences. Hum Gene Ther Methods.

[52] Moorwood C (2013). Isometric and eccentric force generation assessment of skeletal muscles isolated from murine models of muscular dystrophies. J Vis Exp.

[53] Brooks SV, Faulkner JA (1988). Contractile properties of skeletal muscles from young, adult and aged mice. J Physiol.

[54] Ulmer CZ (2015). Liquid chromatography-mass spectrometry metabolic and lipidomic sample preparation workflow for suspension-cultured mammalian cells using Jurkat T lymphocyte cells. J Proteomics Bioinform.

[55] Xu P (2009). Systematical optimization of reverse-phase chromatography for shotgun proteomics. J Proteome Res.

[56] Hunt LC (2021). Integrated genomic and proteomic analyses identify stimulus-dependent molecular changes associated with distinct modes of skeletal muscle atrophy. Cell Rep.

