# Supplementary material for: Targeted Bmal1 restoration in muscle prolongs lifespan with systemic health effects in aging model
Source: JCI Insight. 2024 Oct 1;9(22):e174007. doi: 10.1172/jci.insight.174007 (PMC11601919; doi:10.1172/jci.insight.174007)
Supplement: Supplemental data [file jciinsight-9-174007-s293.pdf]

Supporting Information for

Targeted Bmal1 restoration in muscle prolongs lifespan with systemic health effects in aging model.

Miguel A. Gutierrez-Monreal, Christopher A. Wolff, Eduardo E. Rijos, Mark R. Viggars, Collin M. Douglas, Vishwajeeth Pagala, Junmin Peng, Liam C. Hunt, Haocheng Ding, Zhiguang Huo, Fabio Demontis, and Karyn A. Esser.

Email: kaesser@ufl.edu

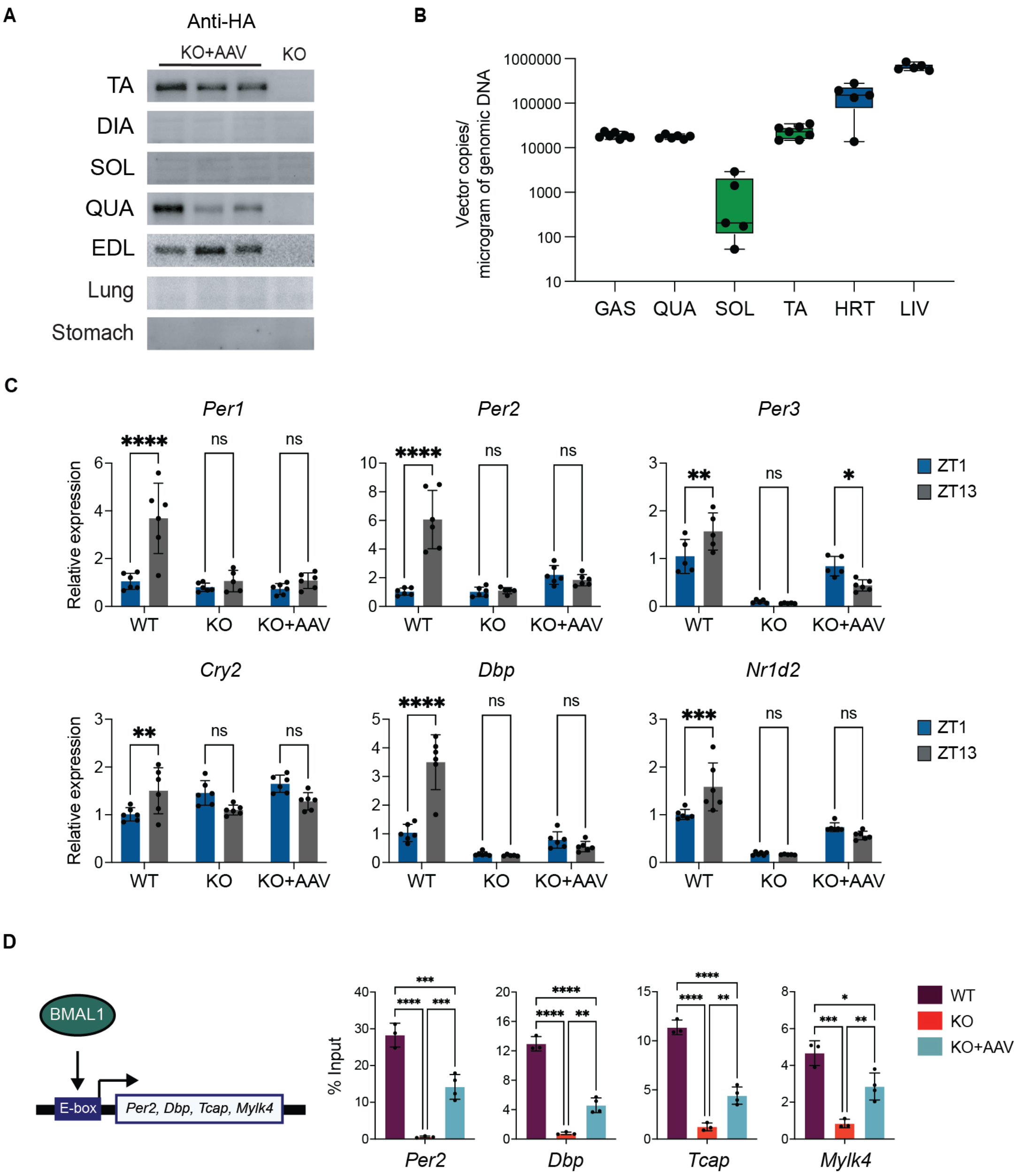

Figure S1. Expression of Bmal1-HA in skeletal muscles and viral genome copy number. A) Western blot for anti-HA detection in tibialis anterior (TA), diaphragm (DIA), soleus (SOL), quadriceps (QUA), and extensor digitorum longus (EDL) in *Bmal1*-KO+AAV mice at 10 weeks of age. Samples from lung and stomach tissues were used as control tissue for non-muscle cells. B) Vector genomes copy numbers in skeletal muscles, heart, and liver (n=5-7 mice). C) Expression of core clock factors and clock output genes at two different time points, ZT1 and ZT13. (n=5-6/group/time point). Two-way ANOVA, *p<0.05, **p < 0.01; ***p < 0.001; ****p < 0.0001. D) ChIP-qPCR using an anti-BMAL1 antibody (n = 3-4/group). One-way ANOVA was used with *p < 0.05, **p < 0.01, ***p < 0.001, ****p < 0.0001.

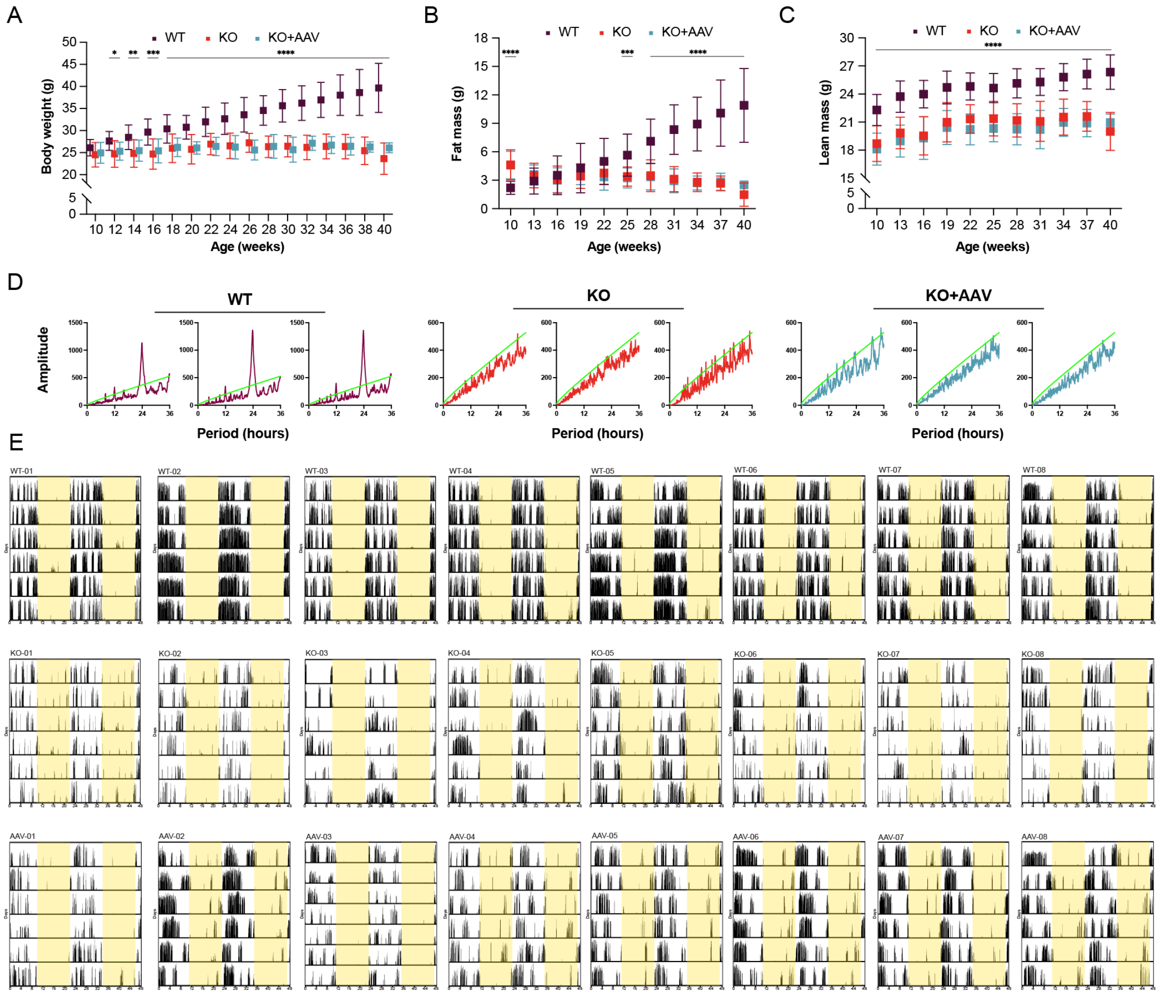

Figure S2. Muscle-specific rescue of *Bmal1* does not alter body composition or behavior. A) Body weight every two weeks from 10 to 40 weeks (n=16-23 mice/group). B-C) Fat and lean mass over time, from 10 to 40 weeks (n=15-23 mice/group). Two-way ANOVA * $p<0.05$, ** $p<0.01$, *** $p<0.001$, **** $p<0.0001$. D) Representative Chi-square periodograms showing no significant periods in the rescue model measured using wheel activity data after two weeks in constant darkness. E) Actograms showing a week of activity recordings (n=8/group).

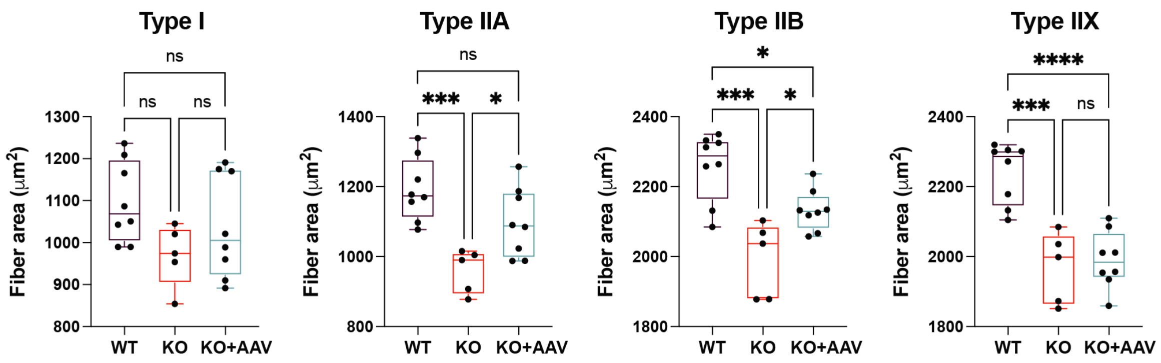

Figure S3. Cross-sectional area of each fiber type. CSA of type I, IIA, IIB, and IIX fibers in WT, KO, and KO+AAV (n=5-8 samples/group). One-way ANOVA, *p<0.05, **p < 0.01, ***p < 0.001; ****p < 0.0001.

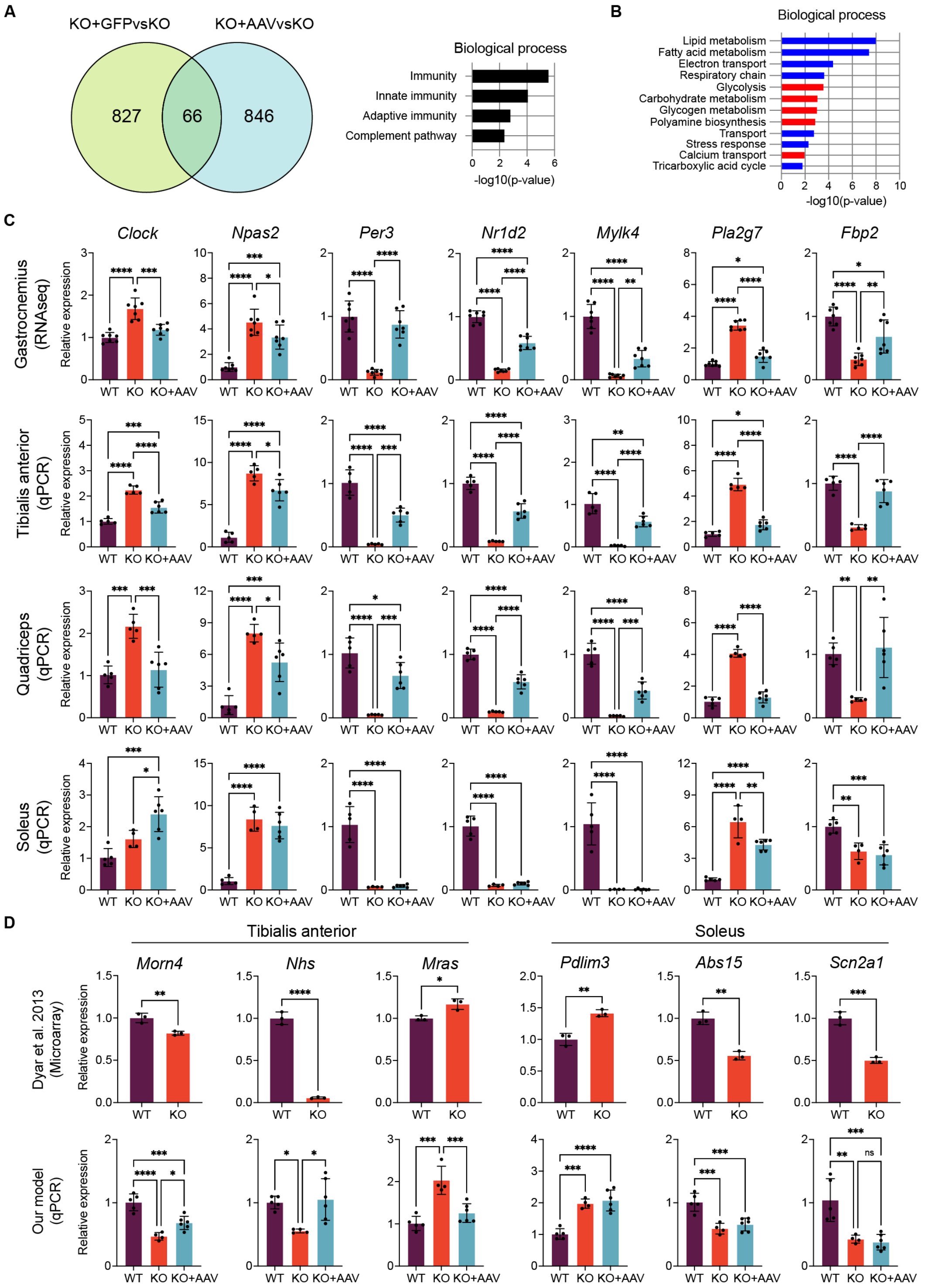

Figure S4. Differentially expressed genes in different skeletal muscles. A) Overlapping analysis of DEGs found in KOvsKO+GFP and KOvsKO+AAV(Bmal1) and biological processes of the overlapping genes. B) Biological processes enriched in DEGs found in KOvsKO+GFP. C) Expression of DEGs found in KO vs KO+AAV gastrocnemius samples measured in TA, quadriceps, and soleus of WT, KO, and KO+AAV (n=5-6 samples/group). One-way ANOVA, *p<0.05, **p < 0.01, ***p < 0.001; ****p < 0.0001. D) Genes from muscle-specific *Bmal1*-KO found dysregulated in TA and soleus by Dyar et al. 2014 (n=5-6 samples/group). One-way ANOVA, *p<0.05, **p < 0.01, ***p < 0.001; ****p < 0.0001.

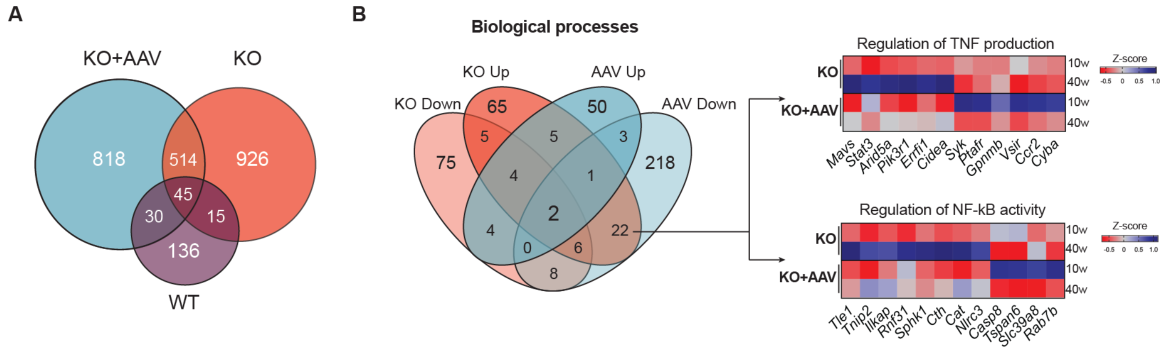

Figure S5. Analysis of genes changing with age. A) Overlap analysis of DEGs in the three groups. B) Venn diagram showing overlapping biological processes enriched in KO+AAV and KO muscle, and heatmap analysis of DEGs enriched in ‘Regulation of TNF production’ and ‘Regulation of NFkB activity.’

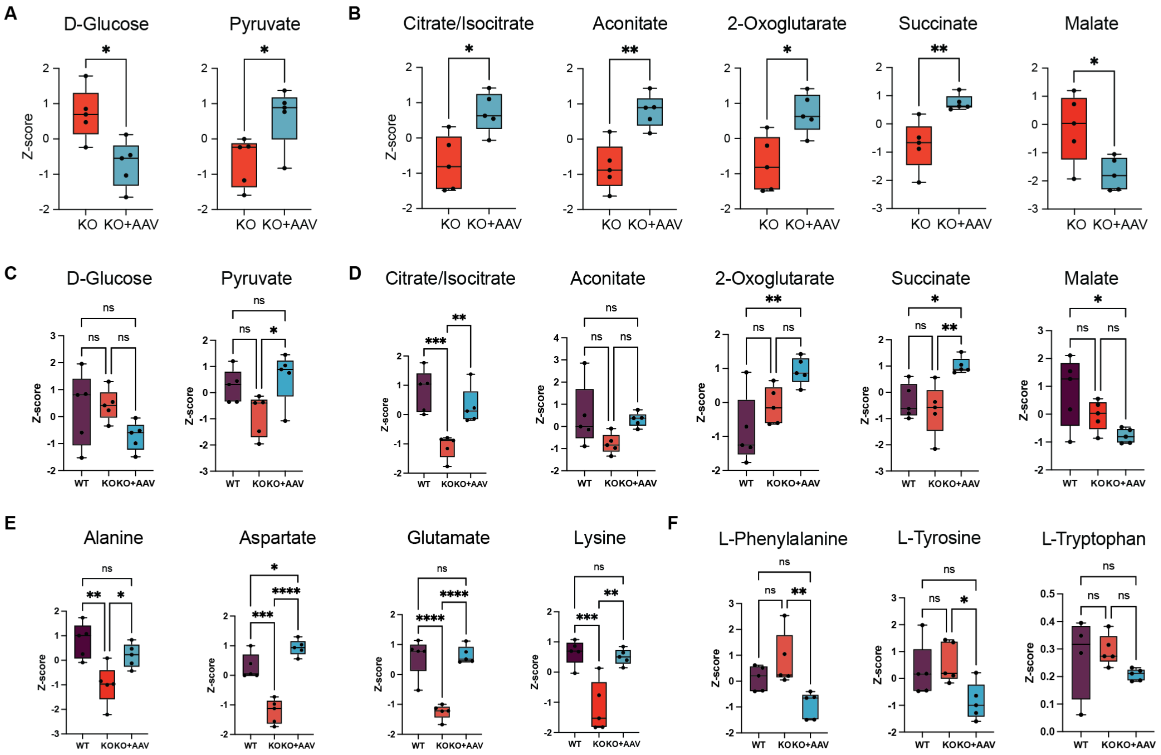

Figure S6. Metabolites from Figure 6H-K with WT values. Metabolites related to A) glycolysis, B) TCA cycle changing in between KO+AAV and KO groups (n = 5 samples/group). Two-tailed Student's, *p < 0.05, **p < 0.01. Metabolites related with C) glycolysis, D) TCA cycle, E) alanine, aspartate, and glutamate metabolism, and F) those associated with inflammatory responses with WT values (n = 5 samples/group). One-way ANOVA, *p < 0.05, **p < 0.01, ***p < 0.001, ****p < 0.0001.

Table S1 – Primers for Chromatin immunoprecipitation assay

Gene symbol	Forward sequence	Reverse sequence	Product size (bp)
Dbp	5'-TGGGACGCCTGGGTACAC-3'	5'-GGGAATGTGCAGCACTGGTT-3'	65
Mylk4	5'-TGCTCGGACTATCCACTCCA-3'	5'-CATAAGGGGACAGCAGGCTC-3'	60
Per2	5'-TCATTTGCATACTGGCGGGG-3'	5'-TATGTAAAGAGAGCGACGGGC-3'	78
Tcap	5'-CAGACACCCAGAGGTGCTAC-3'	5'-AAGCCTGCAAGATGCTCTGT-3'	60

Table S2 – Primers for quantitative real time PCR

Gene symbol	Accession number	Forward sequence	Reverse sequence	Product size
Nr1d1	NM_145434.4	5'-TCCAGTTTGTGTCAAGGTCCA-3'	5'-GGAGCCACTAGAGCCAATGTA-3'	118
Nr1d2	NM_011584.4	5'-AAAGCTGGGACTTTTGAGGTTT-3'	5'-ATCCCCTGCTCCCATTGAGT-3'	129
Clock	NM_007715.6	5'-GTCGAATCTCACTAGCATCTGAC-3'	5'-CTTCCTGGTAACGCGAGAAAG-3'	112
Myod1	NM_010866.2	5'-ATGGATTACAGCGGCCCC-3'	5'-TGTGGAGATGCGCTCCACTA-3'	153
Tcap	NM_011540.2	5'-GATGCGCCTGGGTATCCTC-3'	5'-GATCGAGACAGGGTACGG-3'	273
Mylk4	NM_001368880.1	5'-GCCGAAAAGAATCCTCTACTTGC-3'	5'-AGCACTCCCTTCTCTTGACATCT-3'	82
Gapdh	NM_008084.3	5'-GGAGCCAAACGGGTCATCATCTC-3'	5'-GAGGGGCCATCCACAGTCTTCT-3'	233
Per3	NM_001289877.1	5'-TCAAGACGTGAGGGCGTTCTA-3'	5'-CATTCATACTGCGAGGCTCTTT-3'	90
Cry1	NM_007771.3	5'-CACTGGTTCCGAAAGGGACTC-3'	5'-CTGAAGCAAAAATCGCCACCT-3'	153
Cry2	NM_009963.4	5'-CACTGGTTCCGCAAAGGACTA-3'	5'-CCACGGGTCGAGGATGTAGA-3'	102
Per1	NM_011065.5	5'-CGGATTGTCTATATTTCGGAGCA-3'	5'-TGGGCAGTCGAGATGGTGTA-3'	142
Per2	NM_011066.3	5'-AAAGCTGACGCACACAAAGAA-3'	5'-ACTCCTCATTAGCCTTCACCT-3'	151
Dbp	NM_016974.3	5'-ACCGCTTCTCAGAGGAGGAACTGA-3'	5'-CTTCTTGCATCTCTCGACCTCTTGG-3'	148
Npas2	NM_008719.2	5'-AGGTCATCGGATTCTTGCAGA-3'	5'-GTGTGATACTGTCGGACACATAG-3'	192
Rora	NM_013646.2	5'-GTGGAGACAAATCGTCAGGAAT-3'	5'-TGGTCCGATCAATCAAACAGTTC-3'	135
Pla2g7	NM_013737	5’-CTTTTCACTGGCAAGACACATCT-3’	5’-CGACGGGGTACGATCCATTTC-3’	132
Fbp2	NM_007994	5’-ACCCTGACCCGTTACGTTATG-3’	5’-ACATTCACGCTCCCCGAAATC-3’	161
Morn4	NM_198108	5’-GGCAGAGTAGACGGTTTTGG-3’	5’-GTTCTCGAACAGACCTTCGTT-3’	81
Nhs	NM_001081052	5’-GCTCATGTTAGACCTGTGCG-3’	5’-GTGAGTAAGCTGGATGTCGCT-3’	127
Mras	NM_008624	5’-TGTTCCAAGTGAAAACCTTCCC-3’	5’-GGGTCGTAGTCAGGCACAAA-3’	117
Pdlim3	NM_016798	5’-TGGGGGCATAGACTTCAATCA-3’	5’-CTCCGTACCAAAGCCATCAATAG-3’	121
Abs15	NM_080847	5’-TGAAGCGAGCCAGGCTATATT-3’	5’-AGCTCAAAAATGCGACCTTGC-3’	102
Scn2a1	NM_001099298	5’-ATTTTCGGCTCATTCTTCACACT-3’	5’-GGGCGAGGTATCGGTTTTTGT-3’	176
