# Supplementary material for: Targeted Bmal1 restoration in muscle prolongs lifespan with systemic health effects in aging model
Source: JCI Insight. 2024 Oct 1;9(22):e174007. doi: 10.1172/jci.insight.174007 (PMC11601919; doi:10.1172/jci.insight.174007)

anti-HA

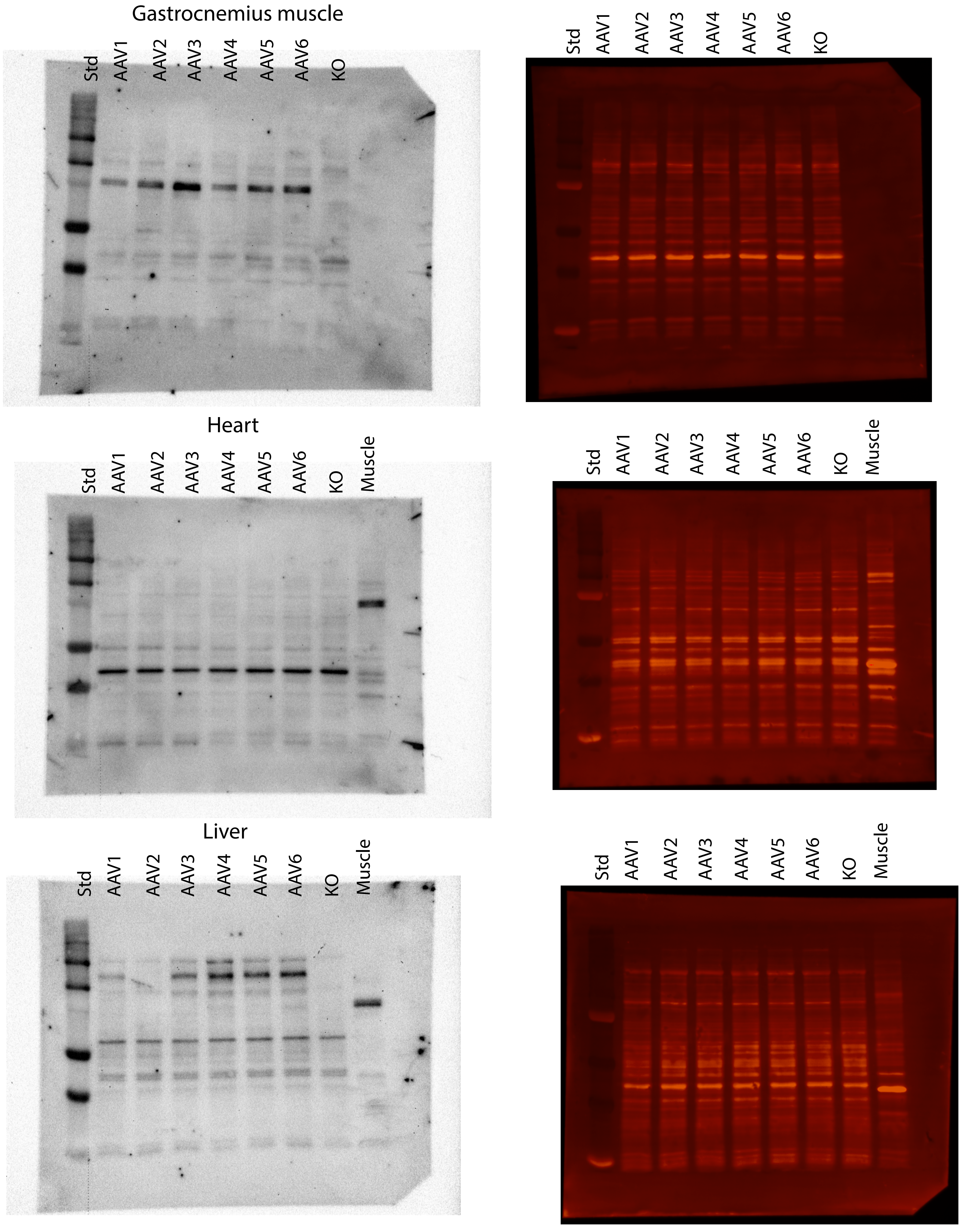

Gastrocnemius muscle
Std AAV1 AAV2 AAV3 AAV4 AAV5 AAV6 KO
Std AAV1 AAV2 AAV3 AAV4 AAV5 AAV6 KO
Heart
Std AAV1 AAV2 AAV3 AAV4 AAV5 AAV6 KO Muscle
Std AAV1 AAV2 AAV3 AAV4 AAV5 AAV6 KO Muscle
Liver
Std AAV1 AAV2 AAV3 AAV4 AAV5 AAV6 KO Muscle
Std AAV1 AAV2 AAV3 AAV4 AAV5 AAV6 KO Muscle

Gastrocnemius anti-BMAL1

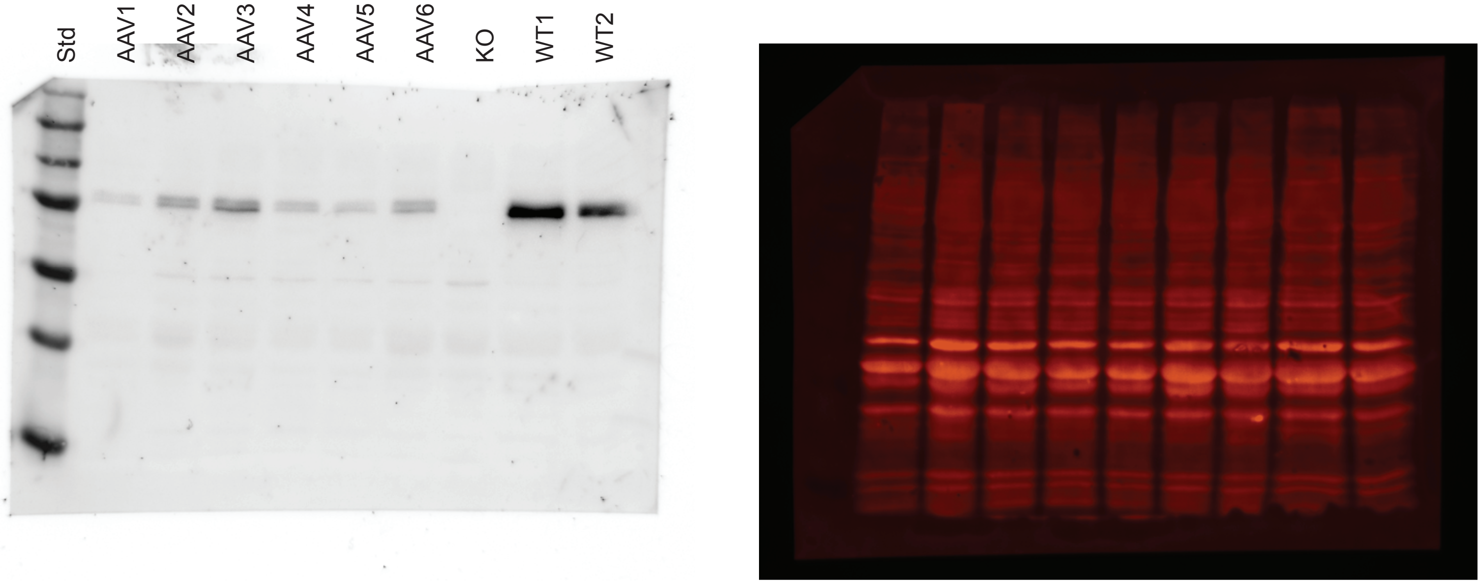

Heart anti-BMAL1

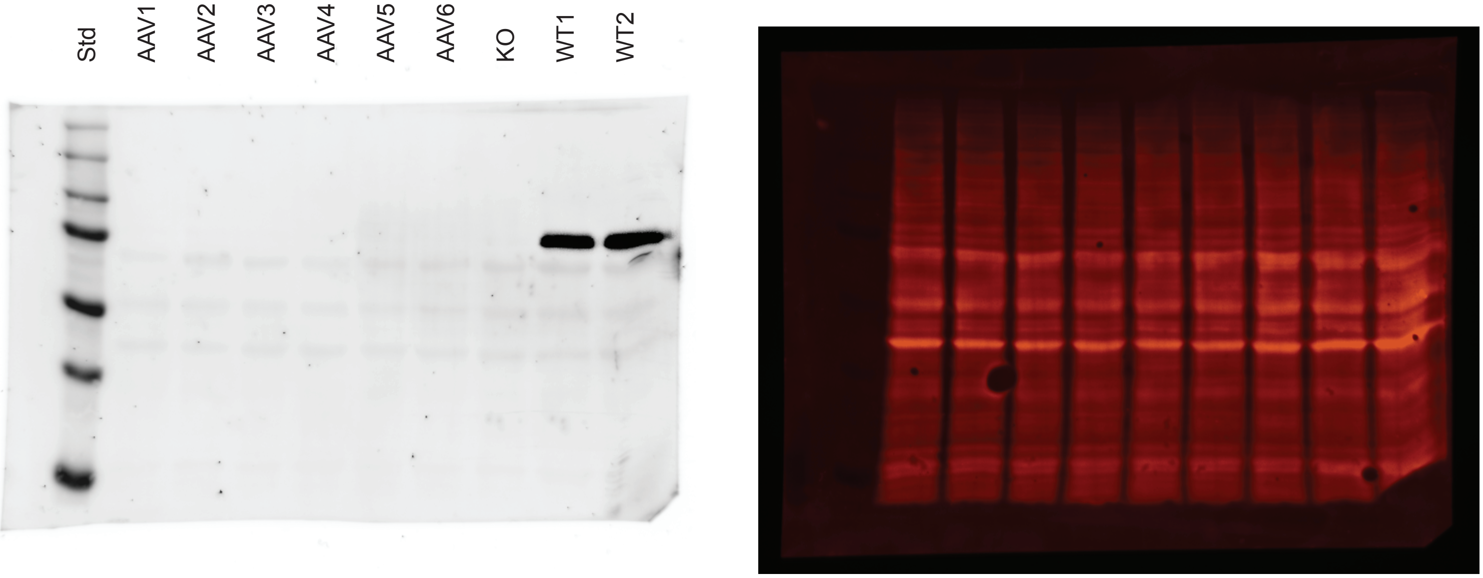

Liver anti-BMAL1

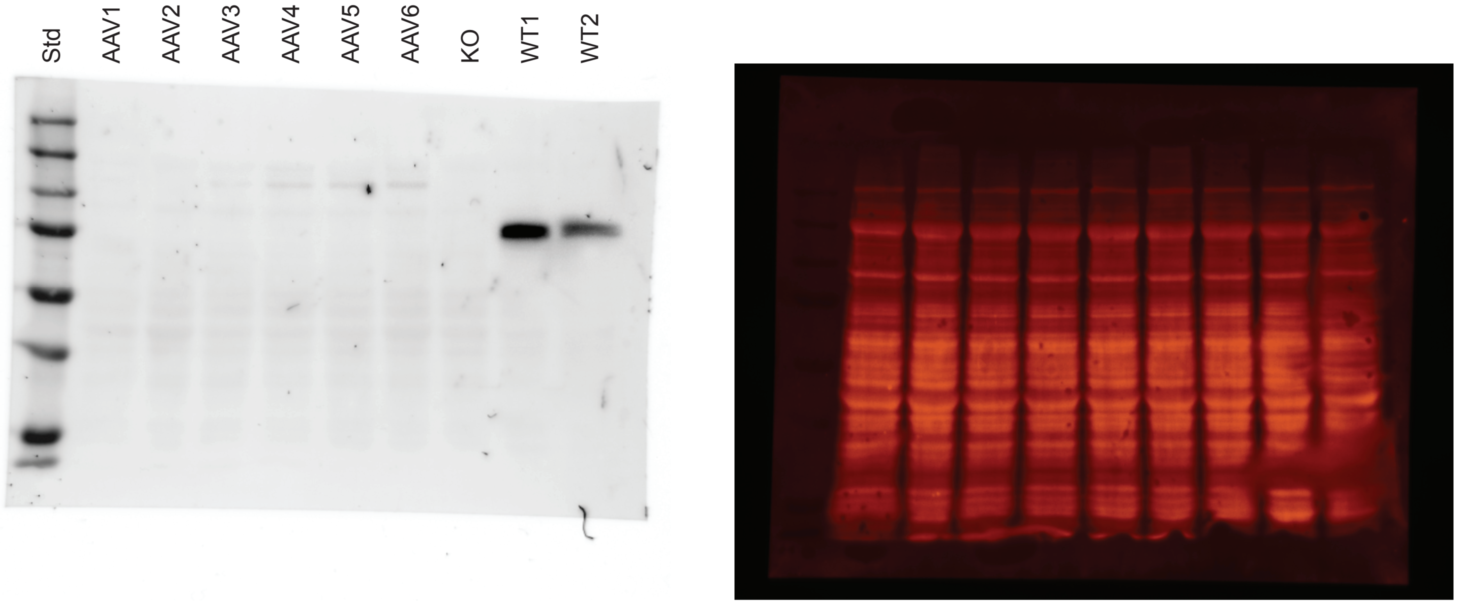

Tibialis anterior (TA) anti-HA

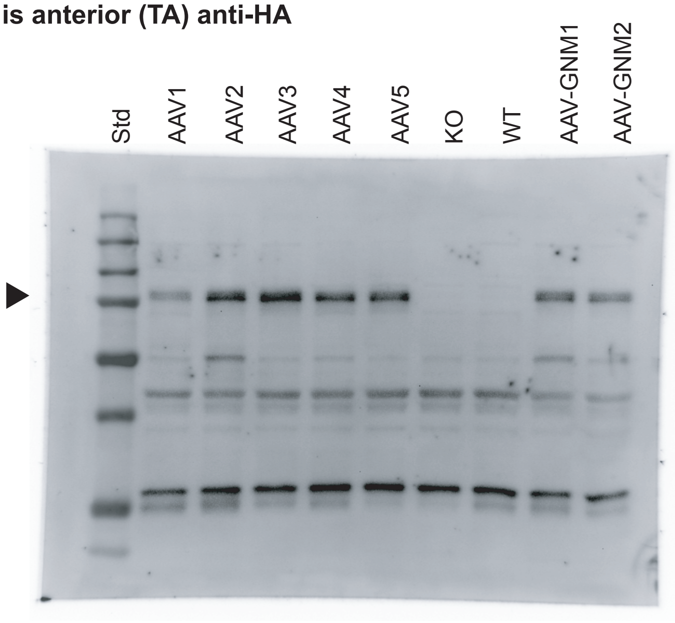

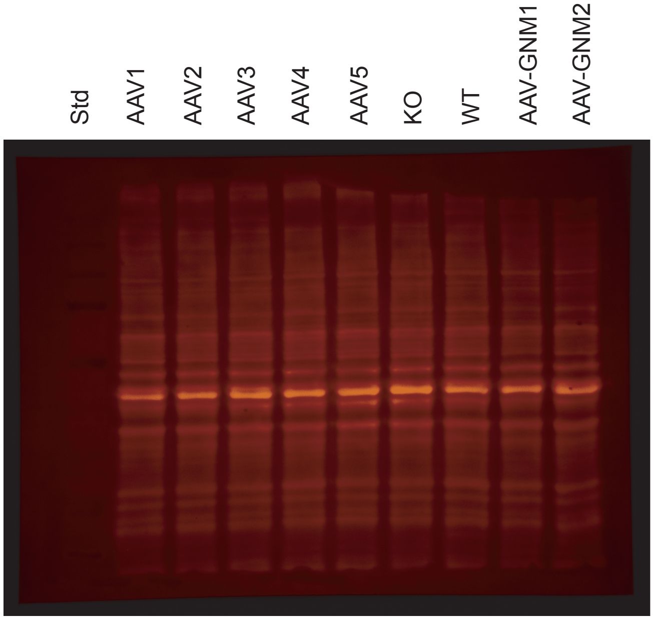

Extensor digitorum longus (DIA) anti-HA

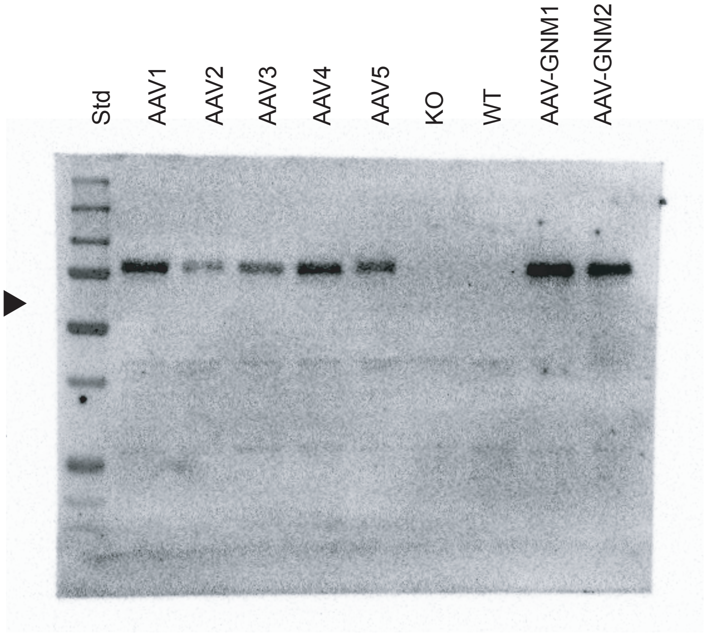

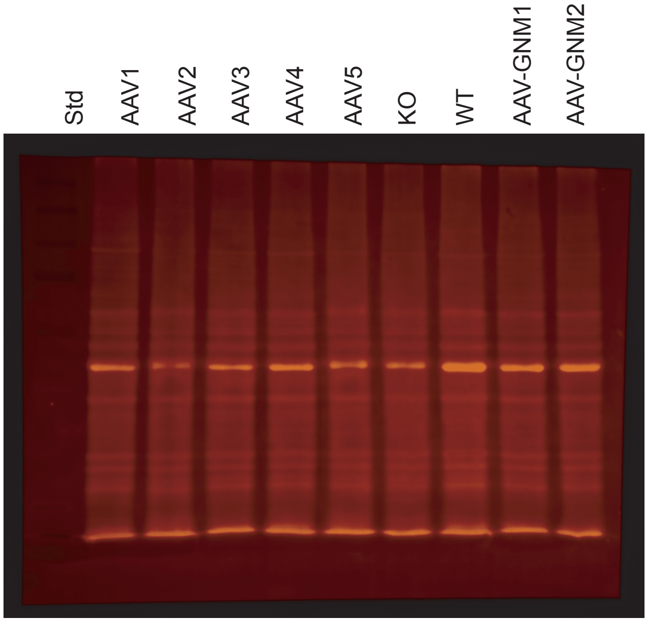

Soleus (SOL) anti-HA

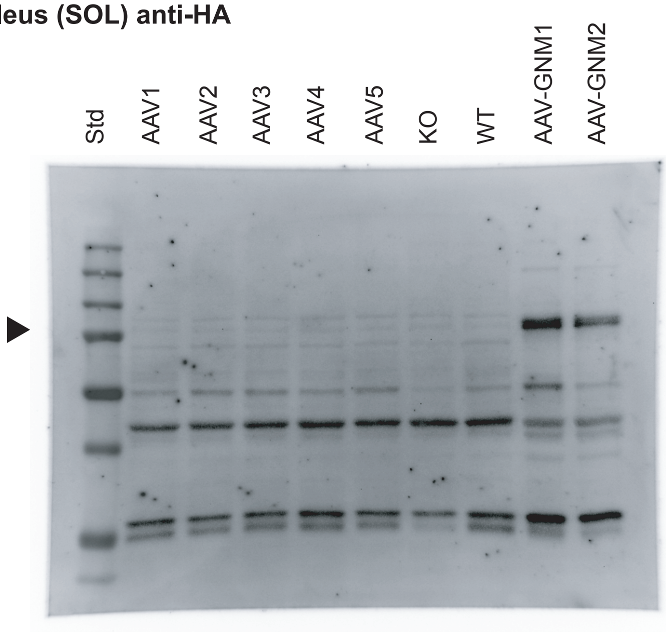

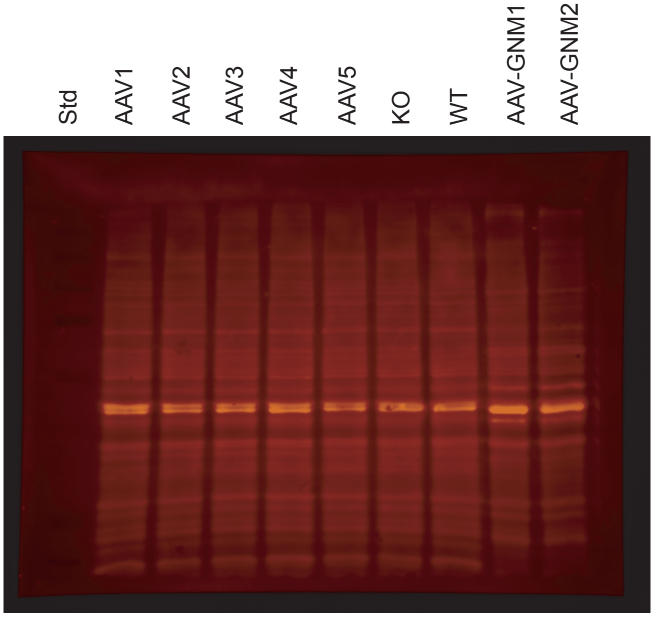

Quadriceps (QUA) anti-HA

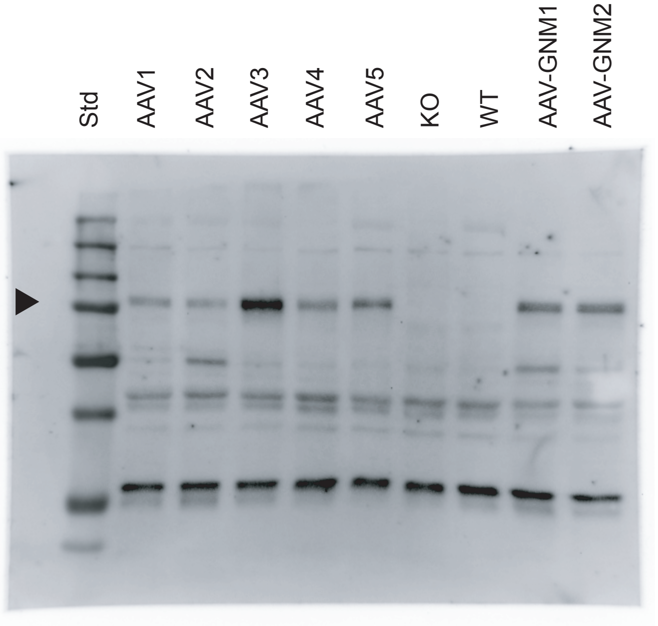

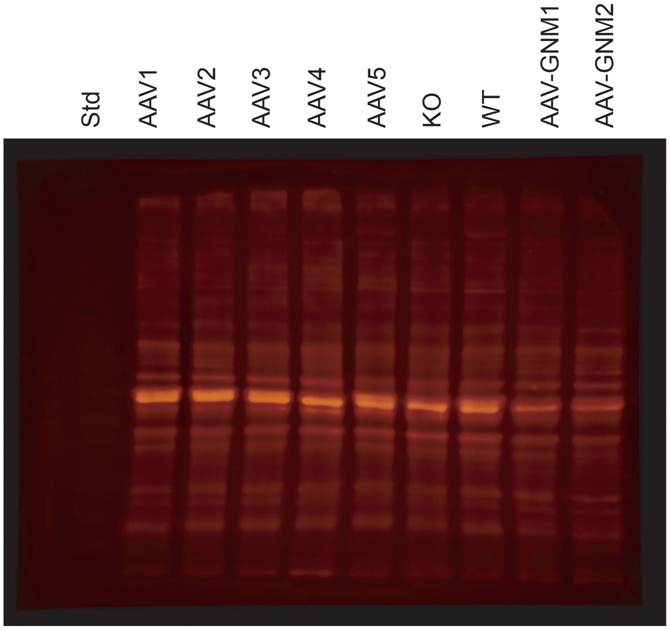

Stomach anti-HA

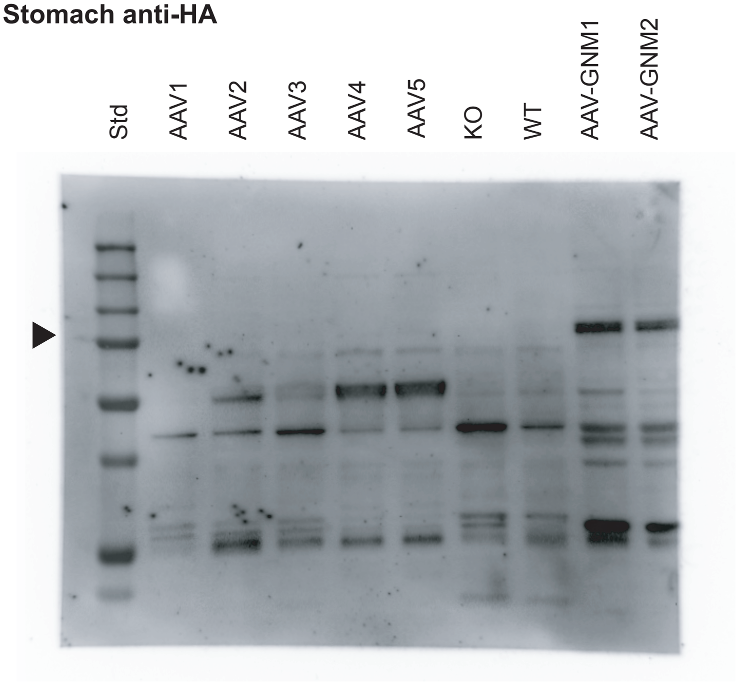

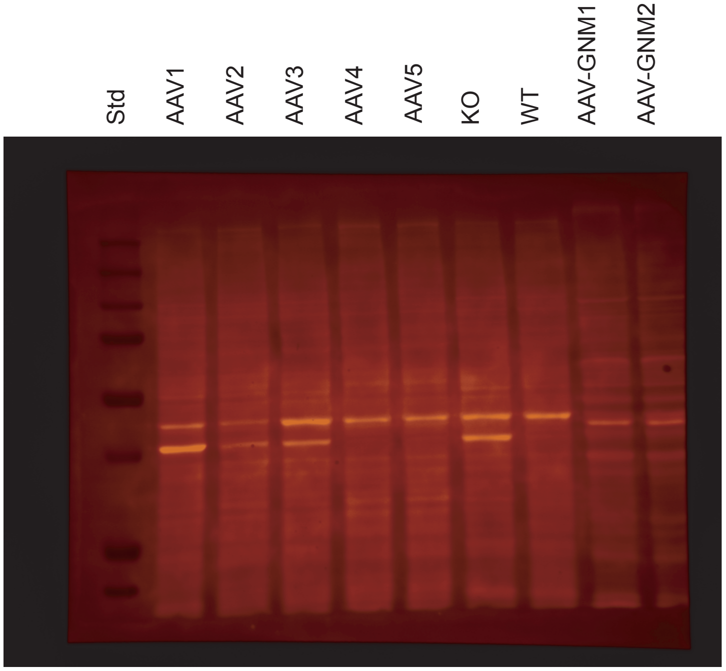

Lung anti-HA

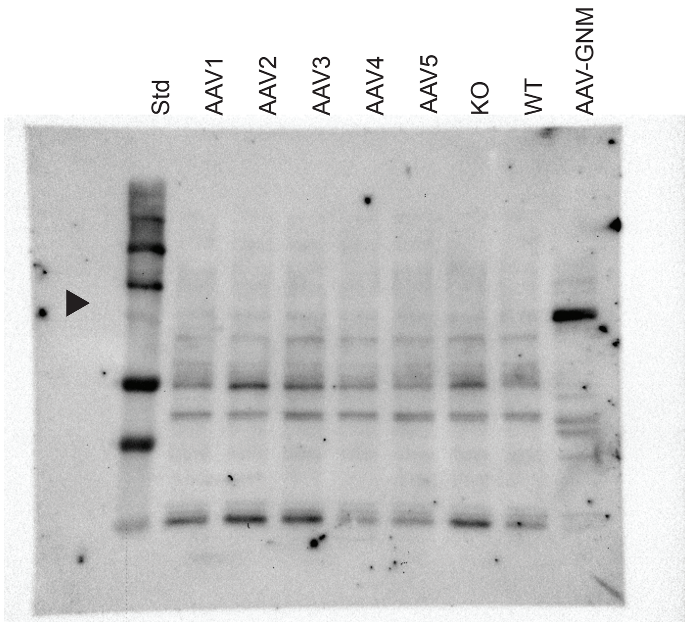

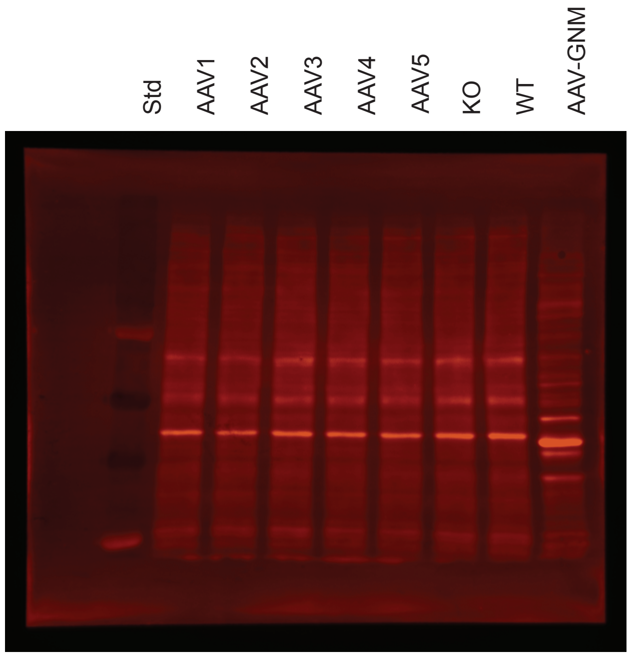

Supplement: Unedited blot and gel images [file jciinsight-9-174007-s296.pdf]
